# Supplementary material for: Cohort profile: rationale and methods of UK Biobank repeat imaging study eye measures to study dementia
Source: BMJ Open. 2023 Jun 23;13(6):e069258. doi: 10.1136/bmjopen-2022-069258 (PMC10314584; doi:10.1136/bmjopen-2022-069258)
Supplement: Author's manuscript [file bmjopen-2022-069258.draft_revisions.pdf]

BMJ Open is committed to open peer review. As part of this commitment we make the peer review history of every article we publish publicly available.

When an article is published we post the peer reviewers' comments and the authors' responses online. We also post the versions of the paper that were used during peer review. These are the versions that the peer review comments apply to.

The versions of the paper that follow are the versions that were submitted during the peer review process. They are not the versions of record or the final published versions. They should not be cited or distributed as the published version of this manuscript.

BMJ Open is an open access journal and the full, final, typeset and author-corrected version of record of the manuscript is available on our site with no access controls, subscription charges or pay-per-view fees (http://bmjopen.bmj.com).

If you have any questions on BMJ Open's open peer review process please email info.bmjopen@bmj.com

BMJ Open

Cohort Profile: Rationale and Methods of UK Biobank Repeat Imaging Study Eye Measures

Journal:	*BMJ Open*
Manuscript ID	bmjopen-2022-069258
Article Type:	Cohort profile
Date Submitted by the Author:	14-Oct-2022
Complete List of Authors:	Foster, Paul; NIHR Biomedical Research Centre at Moorfields Eye Hospital NHS Foundation Trust and UCL Institute of Ophthalmology<br>Atan, Denize; University of Bristol<br>Khawaja, Anthony; 1. NIHR Biomedical Research Centre at Moorfields Eye Hospital NHS Foundation Trust & UCL Institute of Ophthalmology, London, UK<br>Lotery, Andrew; University of Southampton<br>MacGillivray, Tom; Queens Medical Research Institution, Clinical Research Imaging Centre<br>Owen, Christopher; St George's, University London, Population Health Research Institute<br>Patel, Praveen; Moorfields Eye Hospital NHS Foundation Trust, NIHR Biomedical Research Centre<br>Petzold, Axel; Queen Square Institute of Neurology, UCL, Department of Molecular Neurosciences, Moorfields Eye Hospital and The National Hospital for Neurology and Neurosurgery; Amsterdam UMC, Departments of Neurology, Ophthalmology and Expertise Center for Neuro-ophthalmology<br>Rudnicka, Alicja; SGUL University of London<br>Sun, Zihan; University College London, Institute of Ophthalmology<br>Sheard, Simon; UK Biobank<br>Allen, Naomi; University of Oxford, Nuffield Department of Population Health; UK Biobank
Keywords:	OPHTHALMOLOGY, EPIDEMIOLOGY, Dementia < NEUROLOGY

SCHOLARONE™
Manuscripts

I, the Submitting Author has the right to grant and does grant on behalf of all authors of the Work (as defined in the below author licence), an exclusive licence and/or a non-exclusive licence for contributions from authors who are: i) UK Crown employees; ii) where BMJ has agreed a CC-BY licence shall apply, and/or iii) in accordance with the terms applicable for US Federal Government officers or employees acting as part of their official duties; on a worldwide, perpetual, irrevocable, royalty-free basis to BMJ Publishing Group Ltd ("BMJ") its licensees and where the relevant Journal is co-owned by BMJ to the co-owners of the Journal, to publish the Work in this journal and any other BMJ products and to exploit all rights, as set out in our licence.

The Submitting Author accepts and understands that any supply made under these terms is made by BMJ to the Submitting Author unless you are acting as an employee on behalf of your employer or a postgraduate student of an affiliated institution which is paying any applicable article publishing charge ("APC") for Open Access articles. Where the Submitting Author wishes to make the Work available on an Open Access basis (and intends to pay the relevant APC), the terms of reuse of such Open Access shall be governed by a Creative Commons licence – details of these licences and which Creative Commons licence will apply to this Work are set out in our licence referred to above.

Other than as permitted in any relevant BMJ Author's Self Archiving Policies, I confirm this Work has not been accepted for publication elsewhere, is not being considered for publication elsewhere and does not duplicate material already published. I confirm all authors consent to publication of this Work and authorise the granting of this licence.

For peer review only

Cohort Profile: Rationale and Methods of UK Biobank Repeat Imaging Study Eye Measures

Authors:
Paul J Foster[1], Denize Atan[2,3], Anthony P. Khawaja[1], Andrew J. Lotery[4], Tom MacGillivray[5], Christopher G Owen[6], Praveen J. Patel[1], Axel Petzold[7,8], Alicja R. Rudnicka[6], Zihan Sun[1], Simon Sheard[9], Naomi Allen[9,10], *On behalf of UK Biobank and UK Biobank Eye and Vision Consortium*

Affiliations:
1. NIHR Biomedical Research Centre at Moorfields Eye Hospital NHS Foundation Trust and UCL Institute of Ophthalmology, London, UK
2. Bristol Eye Hospital, University Hospitals Bristol & Weston NHS Foundation Trust, Bristol, UK
3. Translational Health Sciences, Bristol Medical School, University of Bristol, Bristol, UK
4. Clinical Neurosciences Group, Clinical and Experimental Sciences, Faculty of Medicine, University of Southampton, Southampton, UK
5. Centre for Clinical Brain Sciences, University of Edinburgh, Edinburgh, UK
6. Population Health Research Institute, St George's, University of London, London, UK
7. Queen Square Institute of Neurology, UCL, Department of Molecular Neurosciences, Moorfields Eye Hospital and The National Hospital for Neurology and Neurosurgery, London, UK
8. Departments of Neurology, Ophthalmology and Expertise Center for Neuro-ophthalmology, Amsterdam UMC, Amsterdam, Netherlands
9. UK Biobank, Stockport, UK
10. Nuffield Department of Population Health, University of Oxford, Oxford, UK

Correspondence to:
Prof Paul J Foster
Address: UCL Institute of Ophthalmology, 11-43 Bath Street, London EC1V 9EL
Email address: p.foster@ucl.ac.uk

For peer review only

ABSTRACT

Purpose: To describe the rationale and methodology of eye and vision assessments in the UK Biobank Repeat Imaging study.

Participants: UK Biobank is a large-scale, multicentre, prospective cohort containing in-depth genetic, lifestyle, environmental and health information from half a million participants aged 40-69 enrolled across the UK. A subset (up to 60,000 participants) of the cohort will be invited to the UK Biobank Repeat Imaging Study to collect repeated brain, cardiac and abdominal magnetic resonance imaging (MRI) scans, whole-body DEXA (Dual-energy x-ray absorptiometry), carotid ultrasound, as well as retinal optical coherence tomography (OCT) and colour fundus photographs.

Findings to date: UK Biobank has helped make significant advances in understanding risk factors for many common diseases, including for dementia and cognitive decline. Ophthalmic genetic and epidemiology studies have also benefited from the unparalleled combination of very large numbers of participants, deep phenotyping, and longitudinal follow-up of the cohort, with comprehensive health data linkage to disease outcomes. In addition, we have used UK Biobank data to describe the relationship between retinal structures, cognitive function and brain MRI-derived phenotypes.

Future plans: UK Biobank is one of the largest prospective cohorts worldwide with extensive data on ophthalmic diseases and conditions. The collection of eye-related data (e.g. OCT), as part of the UK Biobank Repeat Imaging study, will take place between 2022-2028. The depth and breadth and longitudinal nature of this dataset, coupled with its open-access policy, will create a major new resource for dementia diagnostic discovery and to better understand its association with co-morbid diseases.

For peer review only

STRENGTHS AND LIMITATIONS OF THIS STUDY

Strengths

- World's largest prospective, longitudinal multi-modal imaging cohort with unprecedented power for analysis of determinants of a wide range of health outcomes.
- Exceptional added value from the size, depth, and quality of the cross-sectional and longitudinal MRI data on the eye, brain, body and imaging of heart, carotids, together with linkage to electronic health records, through which overt dementia and Alzheimer's disease can be identified.
- Optimal timing to study cognitive impairment (age distribution: ~80% ≥ 60 years and ~65% ≥ 65 years)

Limitations

- ○ Consistency of measurements between imaging devices over time, particularly with use of different OCT devices.
- ○ Healthier participants compared to the general population.

For peer review only

INTRODUCTION

Dementia refers to a heterogeneous group of neurodegenerative disorders affecting 46.8 million people globally.[1-3] Alzheimer's disease (AD) is the commonest, affecting 60-80% of people with dementia.[4, 5] Usually, a long prodromal period of up to 20 years of progressive cerebral atrophy is detectable on magnetic resonance imaging (MRI) scans and using body fluid biomarkers for neurodegeneration before AD is diagnosed.[2] These observations lead to a biological, rather than a clinical definition of AD.[6] To date, the majority of candidate drugs for slowing cognitive decline in AD or other dementias have failed in clinical trials[7], probably because they are used too late in the natural history when irreversible, advanced degeneration has already set in.[8, 9] Global rollout of screening and disease progression monitoring strategies for AD based on MRI scans is precluded by their high cost and frequently limited availability. Body fluid biomarkers might provide ways of stratifying or diagnosing dementias but will remain complimentary to structural imaging biomarkers because of their lack of diagnostic specificity and are not recommended as a screening test.[10]

The eye provides insights into the risk or presence of all systemic diseases, including hypertension and diabetes, as well as changes associated with ~~cognitive~~ ageing and neurodegeneration.[11-13] As an alternative to MRI or plasma biomarkers, optical coherence tomography (OCT) offers a rapid, low-cost and non-invasive method for obtaining high-resolution (3-5µm) images of the retina at the back of the eye – the only part of the central nervous system (CNS) that can be visualized directly. The laminated structure of the retina enables the direct monitoring of neurodegeneration at a near cellular level *in vivo* at a resolution that is more detailed than for any other non-invasive, in vivo imaging modalities. There is strong evidence that quantitative OCT measurements are associated with concurrent cognitive impairment and future cognitive decline and dementia.[14, 15] Additionally, OCT

For peer review only

methods may directly monitor related vascular pathology: amyloid microangiopathy affects retinal and choroidal vasculature, as well as that in the cerebrum with AD.[16] Thus, retinal OCT scans offer the means to identify individuals at high risk of developing AD, providing them with opportunities to change their lifestyles or enter drug trials to delay or avert the onset of dementia. OCT scans are also a sensitive way to monitor patients for neurotoxic side-effects of novel drug treatments. The ability to directly measure specific neuronal layers and microvascular characteristics in detail may provide a surrogate outcome marker for the CNS more generally and potentially enhance the power to detect disease much earlier than methods based on clinical history and genetic factors.

Several genetic (e.g. *APOE*), comorbid (e.g. diabetes, hypertension, depression, obesity), and lifestyle factors (e.g. low educational attainment and smoking) have been associated with increased AD risk.[17-19] However, observational epidemiological studies cannot distinguish cause from effect and are vulnerable to bias from reverse causation and confounders. The analyses by Norton and Larsson illustrate how different disease risk factors can co-exist and are often correlated, but they do not independently increase the risk of AD.[19, 20] It also highlights the importance of identifying causal risk factors for dementia in designing upstream public health policies and social policies to reduce disease risk, clinical trials for new AD drugs and basic science research to understand the underlying mechanisms of dementia development. Using quantitative measurements from OCT scans, it may be possible to assess causal relationships between risk factors and retinal biomarkers related to dementia. The large size of the cohort and the associated healthcare, imaging and genetic data make UK Biobank uniquely valuable for disambiguation of associations from causal comorbidities both for patient stratification and for elucidation of underlying mechanisms.

The research gaps, as mentioned earlier, motivated us to develop a major new resource for dementia diagnostic discovery and to better understand the association with co-morbid

For peer review only

diseases by adding rapid, low-cost OCT to the anticipated UK Biobank Repeat Imaging study, alongside ancillary testing of autorefraction/keratometry and fragmented letter test (FLT).[21] The objectives of this article are to describe (1) the process of test selection (2) the methodology for eye and vision measures in the UK Biobank Repeat Imaging study; (3) the baseline characteristics of the study population in this study.

COHORT DESCRIPTION

UK Biobank

UK Biobank is a large-scale biomedical database and research resource containing in-depth genetic and health information from over 500,000 participants aged 40-69 enrolled across the UK between 2006 and 2010. Detailed study protocols are available on the UK Biobank website (https://www.ukbiobank.ac.uk/). It has become the pre-eminent biomedical research platform for studying the aetiology of common diseases of later life. During the baseline assessment, extensive sociodemographic, lifestyle, and health-related information was collected through a touch screen questionnaire and oral interview, and a wide range of physical measurements was performed.[22, 23] Participants also provided biological samples for genotyping, haematological, biochemistry, metabolomics and proteomics assays for the full cohort.[24]

UK Biobank received approval from the National Information Governance Board for Health and Social Care and the National Health Service Northwest Centre for Research Ethics Committee (Ref: 11/NW/0382). UK Biobank is compliant with the previous Data Protection Act and the more recent General Data Protection Regulation (GDPR) implemented in 2018. For the GDPR, participants were contacted by email or post to explain how UK Biobank meets the requirements of the new regulations (https://www.ukbiobank.ac.uk/gdpr/).

At the baseline assessment in 2006-2010, various eye measures including visual acuity, autorefraction, keratometry, intraocular pressure, corneal biomechanics, and retinal imaging

For peer review only

comprising disc/macular digital colour photographs and a 3D macular OCT were performed on a subset of the UK Biobank participants – e.g., over 110,000 participants have completed the visual acuity, refractive error, and intraocular pressure measurements; and ~67,000 participants underwent retinal imaging. Detailed information on the baseline eye and vision measures has been published elsewhere [22].

The Repeat Imaging Sub-study

In 2014, UK Biobank launched the world's largest multimodal imaging study, intending to include baseline magnetic resonance imaging (MRI) of the brain, heart and abdomen, whole-body DEXA (Dual-energy x-ray absorptiometry) and carotid Doppler ultrasound on up to 100,000 participants. Detailed methods of the UK Biobank imaging enhancement were published elsewhere.[25] Although imaging 100,000 participants is a unique and powerful enhancement to the UKB resource, many valuable insights could only be gained from observing the change in imaging phenotypes over time. Recognising the importance of serial measurements, up to 60,000 of those in the imaging enhancement study will be invited to undergo repeat multimodal imaging between 2019-2028. As part of the repeat imaging study, data collection of the eye measures (e.g., OCT) is anticipated to take place from 2022-2028. The specific study design is as follows:

- All UK Biobank participants who have previously attended a baseline brain and body imaging visit will be invited to attend a repeat imaging visit (the invitation will specify the same imaging centre as their baseline imaging visit to minimize measurement error caused by differences between scanners at different centres).
- Appointment slots are planned in groups of 3 to minimize equipment downtime and to maximize participant throughput and data quality.
- On arrival, those who accept will be asked to consent to the study, and each participant will then undergo a pre-screening safety assessment.

For peer review only

- The 3 participants would then progress to the imaging modalities as follows:
    - Participant #1 => Brain MRI
    - Participant #2 => Abdomen and heart MRI
    - Participant #3 => DeXA, ultrasound and OCT
- Each imaging modality "group" takes approximately 40 minutes, after which the participants will move to the next modality, then switch again after 40 minutes so that each member of the group of 3 has visited all three imaging measurement stations over a 2-hour period.
- The participants then all progress to the non-imaging parts of the visit where they will complete questionnaires, have physical measures, and give biological samples, which mirrors much of the initial (2006-2010) baseline visit.
- As one group of 3 participants exits the imaging part of the visit, the next group of 3 are ready to enter, thus ensuring that the imaging part of the visit is fully utilized.
- This process will repeat for five groups of 3 people (15 total) on 7 days per week at each of UK Biobank's 4 dedicated imaging centres.

Study location

This multisite study will be run from four dedicated UK Biobank Imaging Centres across the UK (Newcastle upon Tyne, Stockport, Reading and Bristol). These 4 centres help ensure most participants are within a reasonable distance to attend a scanning visit. As far as is reasonably practical, maintaining the same instruments and software/firmware across the sites and all phases of the UK Biobank project will ensure consistency and comparability of results from the start of the baseline imaging project to the end of the repeat imaging program. UK Biobank built the following strategies to reduce variability across the different sites:

For peer review only

- The sites are each populated with the same equipment (same manufacturer, same model, same software/firmware etc.) configured with the same protocols and the same settings.
- MR scanner settings/performance across all four sites is monitored by UK Biobank's full-time in-house MR physicist with continuous quality assurance processes to identify and resolve quality issues that may arise.
- All staff are trained to standard operating procedures, and (in the case of the imaging element) compliance/consistency is overseen by an in-house senior radiographer and an in-house MR physicist. The non-imaging aspects are overseen by UK Biobank's dedicated "Training and Monitoring" team.
- Systems are already in place to ensure appropriate levels of training for all operational staff, monitored via Clinic Training Assessments/Training Matrices. These will be extended to cover the OCT measures: appropriate training will be provided, training assessments/matrices extended to cover these measures, and performance monitored.
- Imaging data are routinely made available to members of the project's expert working group, which is made up of experts in each of the imaging modalities; this group monitors the project progress, periodically provides training interventions and critically, periodically/routinely provides an independent view of performance and data output. A similar approach will be taken regarding the OCT measures with data made available to the Eye Consortium members listed in this application for quality control purposes.

Recruitment

The UK Biobank cohort includes a committed and engaged group of participants who are regularly invited for follow up activities: the typical response rate to online surveys is >50%, and there have been very few withdrawals from the study since recruitment (<0.2%). Regular

For peer review only

222 communications with the cohort (via newsletters, participant meetings, study update meetings and the participant section of the UK Biobank website: www.ukbiobank.ac.uk/explore-your-participation) help to maintain enthusiasm for and engagement with the study. Direct telephone communication with individual participants regarding new sub-studies or general participation questions via a dedicated "Participant Contact Centre" (PCC) provides personalized information and reassurance.

228 This study will use the same invitation protocol as the UK Biobank imaging enhancement study (2014-2023)[25]. The planned protocol for this repeat imaging study involves:

230 • E-mail/postal explanation of the study and invitation to book an appointment
231 • A telephone call to book an appointment and perform safety pre-screening via PRC
232 • Assessment at the nearest of four imaging centres across the UK (Stockport, Newcastle, Reading and Bristol) to minimize travel time and maximize participant attendance.

235 At the start of the pandemic (lockdown in the UK in March 2020), 50,000 of the target 100,000 participants had been imaged. Participant questionnaires on completion of baseline imaging visit indicate >90% would be happy to undertake a repeat imaging visit. Pilot studies involving a few thousand participants have demonstrated ~60% acceptance rates, providing confidence that ~60,000 could be recruited for this repeat imaging study.

240 *Examination procedures*

241 The whole-body imaging modalities have been extensively detailed elsewhere[25]; this article only describes the scope of eye and vision measurements in the Repeat Imaging study. The Topcon Triton OCT platform is being used to obtain OCT images in this study. The Triton platform uses ultra-highspeed swept-source (SS) OCT technology with a central wavelength of 1050 microns that penetrates deeper than the retina, allowing visualization of the choroid and the vasculature therein.[26] The platform also takes colour retinal fundus

For peer review only

photographs immediately after the OCT scans, allowing measurement of the optic disc and retinal vessel metrics (including retinal vessel calibre and tortuosity). The Topcon Triton supports wide-angle 12 mm x 9 mm scans that include the optic disc and macula in a single scan.

Widefield SS-OCT enables quantitative measurements of several candidate biomarkers, including but not limited to total macular retinal thickness, macular inner retinal sublayer thicknesses, peripapillary retinal nerve fibre layer thickness, choroidal vascularity index, retinal arteriolar and retinal venular calibres, retinal vascular fractal dimension, retinal vascular tortuosity. Details of the candidate biomarkers are summarized in Table 1.

OCT Image Processing

Total retinal thickness and segmented values for retinal sublayer thicknesses for macula and optic nerve scans are generated by the current generation OCT devices, using FDA approved algorithms, during the examination. In contrast to the processing of baseline UK Biobank macular OCT scans[27], they do not generally require the development of new processing pipelines (apart from measures of the choroidal vascular layer, which are now possible thanks to greater depth of imaging than was previously possible with older OCT technology).

Fundamental to both the challenge and the opportunity that would be provided by UK Biobank OCT imaging is that modern retinal imaging software can measure changes that would be imperceptible to, or missed by, a human grader. In operations featuring large-scale data collection, a small proportion of the imaging is likely to be insufficient quality for automated analysis. Problems may also arise with image acquisition, for instance, due to some study participants presenting with ocular pathology. Thus, the first step in an analysis pipeline is to assess the image quality and discard images that cannot be adequately measured. Subsequent analysis of vasculometrics from retinal photographs would include automated vessel segmentation followed by classification of arterioles and venules (see

For peer review only - http://bmjopen.bmj.com/site/about/guidelines.xhtml

below). For OCT, algorithms delineate the borders of the internal limiting membrane (ILM) and the RNFL to give the measurement of RNFL thickness, a biomarker of axonal loss affecting retinal ganglion cells and the optic nerves. The thickness of the RNFL is evaluated using the standard TSNIT (temporal, superior, nasal, inferior, temporal) mapping that subdivides the measurements and colour codes statistical significance compared with a database of normal healthy values. Further delineation of boundaries enables quantitative mapping of the ganglion cell layer (GCL) and inner plexiform layer (IPL) thicknesses, a marker of neuronal somatic loss [13, 28].

Although the processing of quantitative retinal vasculometric data is not routinely used in clinical settings, we have developed and validated a fully automated AI-enabled retinal image analysis system (QUARTZ) for extracting vessel maps and quantifying retinal vasculometry (including vessel size and tortuosity), which we will use to create the image processing pipeline. The system overcomes many of the difficulties of earlier vasculometry approaches, particularly by being fully automated.[29, 30] QUARTZ has been demonstrated to be highly robust, capable of processing large datasets with automated image quality assessments, resulting in accurate, reliable and high levels of vessel segmentation. To date, QUARTZ has measured approximately 4 million vessel segments from over 190,000 images from 95,000 participants of two very large population-based cohorts (UK Biobank and EPIC-Norfolk). This system has been developed specifically for use on TOPCON macular centred images.

In brief, the QUARTZ system distinguishes between right and left eyes, venules and arterioles (with 87% accuracy using AI-enabled deep learning), identifies vessel segments and centreline coordinates and outputs measures of vessel width and tortuosity (based on the mean change in chord length between successive divisions of the vessel).[31, 32] The system obtains 10-20 thousands of measurements of width and tortuosity from the whole retinal image (dependent on image quality), not just selected vessels lying within concentric areas

For peer review only

For peer review only - http://bmjopen.bmj.com/site/about/guidelines.xhtml

centred on the disc. Measures are summarized using mean width and tortuosity, weighted by segment length, for arterioles and venules separately for each image. QUARTZ measures in UK Biobank have previously shown that venular width and tortuosity are associated with markers of adiposity[33] and that both arteriolar and venular width and arteriolar tortuosity show strong inverse associations with blood pressure (systolic and diastolic) and arterial stiffness index.[34] More importantly, prognostic models using QUARTZ vasculometry measures perform very well at predicting circulatory mortality and at least as well as established risk scores in the prediction of stroke and myocardial infarction, remarkably without the need for either a blood test or blood pressure measurement.[35] Given the identification of vessel maps, these could be inputted into other systems (i.e., the VAMPIRE system) with additional vasculometry summaries, such as fractal analyses to quantify the complexity of the arteriolar and venular components of the retinal vascular network. Marrying the automated functionality of QUARTZ with VAMPIRE will afford a more in-depth characterization of the vessel complex on an unprecedented scale.

The VAMPIRE (Vascular Assessment and Measurement Platform for Images of the REtina) system is an international collaborative project designed to quantify retinal vascular morphometry with large collections of fundus photographs. The system provides automatic detection of retinal landmarks and quantifies some key parameters used frequently in investigative studies - vessel width, vessel branching coefficients, tortuosity, and fractal analyses. Detailed definitions have been reported elsewhere.[36, 37] In general, it computes 149 measurements per image, including basic statistics. Thirty-nine are width-related: central retinal arteriolar equivalent (CRAE), central retinal venular equivalent (CRVE), retinal arteriovenous ratio (AVR), basic statistics (mean, median, standard deviation, maximum, minimum), width gradients along vessels, average ratio length-diameter at branching points, by arteries and veins; 104 are tortuosity measurements, computed by different algorithms and

For peer review only

322 with the statistics listed above; 6 are fractal dimension coefficients. All measures are
323 calculated by vessel type (arteriole or venule) and region (zone, whole image, quadrants).
324 VAMPIRE is a validated software application and has been extensively used in several
325 international studies.[36, 38, 39]

326 *Patient and public involvement*

327 UK Biobank maintains a website to keep participants and researchers up to date on the study
328 (http://www.ukbiobank.ac.uk/news/). Eye and vision-related publications resulting from UK
329 Biobank are maintained at (https://www.ukbiobankeyeconsortium.org.uk/publications). UK
330 Biobank also holds regular events to inform the participants about the imaging study and the
331 latest research. In addition, UK Biobank has a Twitter feed (@uk_biobank). The study was
332 set up by the Medical Research Council (MRC), Department of Health (DoH), and Wellcome
333 Trust with input from major patient representative organizations. An annual scientific
334 meeting is recorded and available to the public as a webcast.

335 *Statistical Analysis Plan*

336 Baseline ocular characteristics will be summarized as mean (standard deviation) for
337 continuous variables and number (%) for categorical variables.
338 Primary aims would be to examine:
339 1) cross-sectional associations between retinal biomarkers, measures of cognitive
340 performance and brain-volume from MRI imaging
341 2) the comparative performance of retinal biomarkers for risk stratification, to identify
342 those with cognitive impairment
343 3) the comparative performance of retinal biomarkers to detect those with longitudinal
344 decline in cognitive performance

For peer review only

Previous work within UK Biobank examining RNFL measures in relation to mild cognitive impairment, showed that those in the lowest quintile of RNFL thickness were 11% (95%CI: 2% to 21%) more likely to fail on at least one of four cognitive tests.[40] This shows that RNFL measures have the potential to identify those at higher risk of cognitive impairment. After vigorous image quality control, the proposed imaging of a further 60,000 participants will provide 45,000-55,000 participants with good-quality retinal images for quantification of individual components of the RNFL and potential to extract detailed retinal vasculometric measures. This large sample size, will have 99% power (alpha = 0.001) to detect at least 0.03 standard deviation change in the cognitive score[41] or brain measures[42] per 1 standard deviation increase in any retinal biomarker (RNFL or retinal vasculometric measure). Cross-sectional analyses using multiple linear regression will quantify the dose response relationship between cognitive score with considerable power to evaluate in the region of 30 candidate predictors (retinal biomarkers, age, sex, geographical location, height, refraction, intraocular pressure, smoking status, socioeconomic positions and established cardiovascular risk markers).[43] This will allow the independent contribution of retinal biomarkers as a predictor of cognitive performance to be realized with considerable precision,[44] across a spectrum of cognitive scores.[43]

Given that UK Biobank has longitudinal data on cognitive change (with repeated measures available from online questionnaires and performed in-person at the imaging assessments), the study would be uniquely placed to assess the determinants of cognitive decline in middle-later life. For prospective evaluation the rates of dementia would also be pivotal in relation to prior cognitive performance. In UK Biobank, the annual incidence of dementia among those aged ≥ 60 years old is approximately 2.5 per 1000 person years.[45] Therefore, within 2 years of retinal image capture there would be approximately 250 cases of dementia per 45,000-55,000 participants.

For peer review only

Existing Data

Once recruitment was fully under way, additional measures were incorporated into the baseline assessment, including hearing and arterial stiffness tests, a cardiorespiratory fitness test, and various eye and vision measures, including visual acuity on a computerised system designed to observe logMAR principles, and following the British Standard (BS-1968),[46] autorefraction and keratometry, intraocular pressure and corneal biomechanics, and retinal imaging comprising disc/macular digital colour photographs and a 3D macular OCT.[22] After the baseline visit, subsets of participants have supported additional data collection through various enhancements to the study. These have included: a complete repeat of the baseline assessment, collection of physical activity data over 7-days by wearing accelerometers, and regular online questionnaires covering various topics such as diet, cognitive function, occupational history, mental wellbeing, gastrointestinal health and pain. All participants provided consent for their health to be followed-up through linkage to health-related records, which currently includes death, cancer, and hospital inpatient records for the entire cohort. Although UK Biobank is not representative of the entire UK population, the large sample size and variation across all levels of measures nonetheless enable a valid assessment of many exposure-outcome relationships to be made. All publications using UK Biobank data are available on the website (https://www.ukbiobank.ac.uk/enable-your-research/publications). Eye and vision-related publications resulting from UK Biobank is maintained at (https://www.ukbiobankeyeconsortium.org.uk/publications).

Baseline characteristics of the study participants were summarized in Table 2. In addition to imaging, UK Biobank has implemented a wide range of cognitive function tests since baseline that are relevant to assessing various aspects of cognitive decline and dementia and will be conducted at the repeat imaging and proposed OCT visit (Table 3).

FINDINGS TO DATE

For peer review only

UK Biobank has helped make significant advances in the understanding of risk factors for diseases including cardiovascular diseases, cancer, diabetes, stroke, multiple sclerosis, optic neuritis and dementia.[23, 47-57] Ophthalmic genetics and epidemiology have benefited from the unparalleled combination of very large numbers of participants, very extensive and detailed phenotyping and longitudinal follow-up.[30, 58-62]

In addition, we have used UK Biobank data to describe the relationship between retinal structures and both cognitive function and brain MR image-derived phenotypes.[40, 42] For example, previous work examining RNFL measures in relation to mild cognitive impairment, showed that those in the lowest quintile of RNFL thickness were 11% (95% CI 2.0% to 2.1%) more likely to fail on at least one of four cognitive tests.[40] This indicates that RNFL thickness measurements have the potential to identify those at higher risk of cognitive impairment. Chua *et al* [42] reported that markers of retinal neurodegeneration are associated with smaller brain volumes – macular ganglion cell-inner plexiform layer (GCIPL) thickness, ganglion cell complex (GCC) thickness and total macular thickness were significantly associated with smaller total brain ($p < 0.001$), grey matter and white matter volume ($p < 0.01$), and grey matter volume in the occipital pole ($p < 0.05$); thinner macular GCC and total macular thicknesses were associated with smaller hippocampal volume ($p < 0.02$).

In the context of these results, and the findings of other studies (e.g. The Rotterdam Study),[63-65] we proposed supplementing the testing menu in the UK Biobank Whole Body Repeat Imaging Study with measures that support the discovery and quantification of eye and vision variables that are associated with cognitive ageing and decline, and overt dementia.

COLLABORATION

UK Biobank aims to provide open access data for healthcare-related research. The data are available to all bona fide researchers from the academic, charity, public and commercial sectors in the UK and internationally, without preferential or exclusive access for any

user.[66] All interested researchers may apply to access the data via an online application. Strict guidelines are in place to help ensure anonymity and confidentiality of participants' data and samples.[67] We have formed the UK Biobank Eye and Vision Consortium, an 80 person strong group of researchers with interest and expertise in ophthalmic epidemiology, visual system neurology, and the epidemiology of related diseases such as diabetes and cardiovascular disease (https://www.ukbiobankeyeconsortium.org.uk/).

FURTHER DETAILS

Acknowledgements:

In addition to the listed authors, Prof Rory Collins, Prof Paul Matthews and Dr Mark Effingham participated in scientific discussions which moulded the project that we have outlined here. A funding proposal was developed following discussions with members of the Alzheimer's Drug Discovery Foundation. Meanwhile, we would like to thank all the participants of UK Biobank for their vital contribution to the resource.

Data sharing statement:

This research used data from the UK Biobank Resource, under data access request number 2112.

Contributorship statement:

PJF, APK, PJP & ZS had full access to all the data in the study and take responsibility for the integrity and accuracy of the data analysis. Concept and design: PJF, DA, APK, AJL, TM, CGO, PJP, AP, ARR. Data acquisition, analysis, or interpretation: UK Biobank obtained the data. APK performed data analysis. All authors interpreted data. Critical revision of the manuscript for important intellectual content: all authors. Obtained funding: NA, SS, UK Biobank. All authors approved the final manuscript.

Funding declaration:

For peer review only

The study sponsor/funder was not involved in the design of the study; the collection, analysis, and interpretation of data; writing the report; and did not impose any restrictions regarding the publication of the report. UK Biobank is funded by the Medical Research Council, Wellcome Trust, Department of Health, Scottish Government, the Welsh Assembly Government, British Heart Foundation, Cancer Research UK, NIHR and the Northwest Regional Development Agency. The UK Biobank Eye and Vision Consortium is supported by grants from Moorfields Eye Charity, the NIHR Biomedical Research Centre at Moorfields Eye Hospital NHS Foundation Trust and UCL Institute of Ophthalmology, the Alcon Research Institute and the International Glaucoma Association (UK).

APK, AP, ZS, PJF and PJP receive salary support from the NIHR BRC at Moorfields Eye Hospital & UCL Institute of Ophthalmology. NA receives salary support from University of Oxford and UK Biobank. PJF receives support from the Desmond Foundation, London, UK. APK is supported by a UKRI Future Leaders Fellowship and an Alcon Research Institute Young Investigator Award. TM acknowledges support from NHS Lothian R&D and the Clinical Research Facility at the University of Edinburgh.

The authors acknowledge a proportion of our financial support from the UK Department of Health through an award made by the National Institute for Health Research to Moorfields Eye Hospital NHS Foundation Trust and UCL Institute of Ophthalmology for a Biomedical Research Centre for Ophthalmology.

Financial disclosures:

PJF reports personal fees from Allergan, Carl Zeiss, Google/DeepMind and Santen, a grant from Alcon, outside the submitted work. PJP reports grants from Topcon Inc, outside the scope of the current report. APK reports personal fees from Abbvie, Aerie, Google Health, Novartis, Reichert, Santen, and Thea, outside the submitted work. AP reports grant support for remyelination trials in multiple sclerosis to the Amsterdam University Medicam Centre,

For peer review only

Department of Neurology, MS Centre (RESTORE trial) and UCL, London RECOVER trial; Fight for Sight (nimodipine in optic neuritis trial); royalties or licenses from Up-to-Date (Wolters Kluver) on a book chapter; speaker fees for the Heidelberg Academy; participation on Advisory Board SC Zeiss OCTA Angi-Network, SC Novartis OCTiMS study; equipment: OCTA from Zeiss (Plex Elite).

For peer review only

REFERENCES

1. Reitz C, Mayeux R: **Alzheimer disease: epidemiology, diagnostic criteria, risk factors and biomarkers**. *Biochemical pharmacology* 2014, **88**(4):640-651.
2. Bateman RJ, Xiong C, Benzinger TL, Fagan AM, Goate A, Fox NC, Marcus DS, Cairns NJ, Xie X, Blazey TM: **Clinical and biomarker changes in dominantly inherited Alzheimer's disease**. *N Engl J Med* 2012, **367**:795-804.
3. Tahami Monfared AA, Byrnes MJ, White LA, Zhang Q: **Alzheimer's Disease: Epidemiology and Clinical Progression**. *Neurol Ther* 2022, **11**(2):553-569.
4. Kalaria RN, Maestre GE, Arizaga R, Friedland RP, Galasko D, Hall K, Luchsinger JA, Ogunniyi A, Perry EK, Potocnik F: **Alzheimer's disease and vascular dementia in developing countries: prevalence, management, and risk factors**. *The Lancet Neurology* 2008, **7**(9):812-826.
5. Collaborators G: **Global, regional, and national burden of Alzheimer's disease and other dementias, 1990-2016: a systematic analysis for the Global Burden of Disease Study 2016**. *Lancet Neurol* 2019, **18**(1):88-106.
6. Jack Jr CR, Bennett DA, Blennow K, Carrillo MC, Dunn B, Haeberlein SB, Holtzman DM, Jagust W, Jessen F, Karlawish J: **NIA-AA research framework: toward a biological definition of Alzheimer's disease**. *Alzheimer's & Dementia* 2018, **14**(4):535-562.
7. Mehta D, Jackson R, Paul G, Shi J, Sabbagh M: **Why do trials for Alzheimer’s disease drugs keep failing? A discontinued drug perspective for 2010-2015**. *Expert opinion on investigational drugs* 2017, **26**(6):735-739.
8. Gómez-Isla T, Price JL, McKeel Jr DW, Morris JC, Growdon JH, Hyman BT: **Profound loss of layer II entorhinal cortex neurons occurs in very mild Alzheimer’s disease**. *Journal of Neuroscience* 1996, **16**(14):4491-4500.
9. Long JM, Holtzman DM: **Alzheimer disease: an update on pathobiology and treatment strategies**. *Cell* 2019, **179**(2):312-339.
10. Dubois B, Villain N, Frisoni GB, Rabinovici GD, Sabbagh M, Cappa S, Bejanin A, Bombois S, Epelbaum S, Teichmann M: **Clinical diagnosis of Alzheimer's disease: recommendations of the International Working Group**. *The Lancet Neurology* 2021, **20**(6):484-496.
11. Cheung CY-l, Ikram MK, Sabanayagam C, Wong TY: **Retinal microvasculature as a model to study the manifestations of hypertension**. *Hypertension* 2012, **60**(5):1094-1103.
12. Lesage S, Mosley T, Wong T, Szklo M, Knopman D, Catellier DJ, Cole S, Klein R, Coresh J, Coker L: **Retinal microvascular abnormalities and cognitive decline: the ARIC 14-year follow-up study**. *Neurology* 2009, **73**(11):862-868.
13. Petzold A, Balcer LJ, Calabresi PA, Costello F, Frohman TC, Frohman EM, Martinez-Lapiscina EH, Green AJ, Kardon R, Outteryck O: **Retinal layer segmentation in multiple sclerosis: a systematic review and meta-analysis**. *The Lancet Neurology* 2017, **16**(10):797-812.
14. O’Bryhim BE, Apte RS, Kung N, Coble D, Van Stavern GP: **Association of preclinical Alzheimer disease with optical coherence tomographic angiography findings**. *JAMA ophthalmology* 2018, **136**(11):1242-1248.

For peer review only

15. Mutlu U, Colijn JM, Ikram MA, Bonnemaijer PW, Licher S, Wolters FJ, Tiemeier H, Koudstaal PJ, Klaver CC, Ikram MK: **Association of retinal neurodegeneration on optical coherence tomography with dementia: a population-based study**. *JAMA neurology* 2018, **75**(10):1256-1263.
16. Masuzzo A, Dinet V, Cavanagh C, Mascarelli F, Krantic S: **Amyloidosis in retinal neurodegenerative diseases**. *Frontiers in Neurology* 2016, **7**:127.
17. Reitz C, Brayne C, Mayeux R: **Epidemiology of Alzheimer disease**. *Nature Reviews Neurology* 2011, **7**(3):137-152.
18. Lambert J-C, Ibrahim-Verbaas CA, Harold D, Naj AC, Sims R, Bellenguez C, Jun G, DeStefano AL, Bis JC, Beecham GW: **Meta-analysis of 74,046 individuals identifies 11 new susceptibility loci for Alzheimer's disease**. *Nature genetics* 2013, **45**(12):1452-1458.
19. Norton S, Matthews FE, Barnes DE, Yaffe K, Brayne C: **Potential for primary prevention of Alzheimer's disease: an analysis of population-based data**. *The Lancet Neurology* 2014, **13**(8):788-794.
20. Larsson SC, Traylor M, Malik R, Dichgans M, Burgess S, Markus HS: **Modifiable pathways in Alzheimer's disease: Mendelian randomisation analysis**. *bmj* 2017, **359**.
21. Bowen M, Zutshi H, Cordiner M, Crutch S, Shakespeare T: **Qualitative, exploratory pilot study to investigate how people living with posterior cortical atrophy, their carers and clinicians experience tests used to assess vision**. *BMJ open* 2019, **9**(3):e020905.
22. Chua SYL, Thomas D, Allen N, Lotery A, Desai P, Patel P, Muthy Z, Sudlow C, Peto T, Khaw PT: **Cohort profile: design and methods in the eye and vision consortium of UK Biobank**. *BMJ open* 2019, **9**(2):e025077.
23. Littlejohns TJ, Sudlow C, Allen NE, Collins R: **UK Biobank: opportunities for cardiovascular research**. *European heart journal* 2019, **40**(14):1158-1166.
24. Bycroft C, Freeman C, Petkova D, Band G, Elliott LT, Sharp K, Motyer A, Vukcevic D, Delaneau O, O'Connell J: **The UK Biobank resource with deep phenotyping and genomic data**. *Nature* 2018, **562**(7726):203-209.
25. Littlejohns TJ, Holliday J, Gibson LM, Garratt S, Oesingmann N, Alfaro-Almagro F, Bell JD, Boultwood C, Collins R, Conroy MC: **The UK Biobank imaging enhancement of 100,000 participants: rationale, data collection, management and future directions**. *Nature communications* 2020, **11**(1):1-12.
26. Huber R, Wojtkowski M, Fujimoto JG, Jiang J, Cable A: **Three-dimensional and C-mode OCT imaging with a compact, frequency swept laser source at 1300 nm**. *Optics express* 2005, **13**(26):10523-10538.
27. Keane PA, Grossi CM, Foster PJ, Yang Q, Reisman CA, Chan K, Peto T, Thomas D, Patel PJ, Consortium UBEV: **Optical coherence tomography in the UK biobank study–rapid automated analysis of retinal thickness for large population-based studies**. *PLoS One* 2016, **11**(10):e0164095.
28. Cruz-Herranz A, Balk LJ, Oberwahrenbrock T, Saidha S, Martinez-Lapiscina EH, Lagreze WA, Schuman JS, Villoslada P, Calabresi P, Balcer L: **The APOSTEL recommendations for reporting quantitative optical coherence tomography studies**. *Neurology* 2016, **86**(24):2303-2309.
29. Welikala R, Fraz M, Habib M, Daniel-Tong S, Yates M, Foster P, Whincup P, Rudnicka AR, Owen CG, Strachan D: **Automated quantification of retinal vessel morphometry**

For peer review only

in the UK Biobank Cohort. In: *2017 Seventh International Conference on Image Processing Theory, Tools and Applications (IPTA): 2017*: IEEE; 2017: 1-6.

30. Welikala R, Fraz M, Foster P, Whincup P, Rudnicka AR, Owen CG, Strachan D, Barman SA: **Automated retinal image quality assessment on the UK Biobank dataset for epidemiological studies**. *Computers in biology and medicine* 2016, **71**:67-76.
31. Fraz MM, Welikala R, Rudnicka AR, Owen CG, Strachan D, Barman SA: **QUARTZ: Quantitative Analysis of Retinal Vessel Topology and size–an automated system for quantification of retinal vessels morphology**. *Expert Systems with Applications* 2015, **42**(20):7221-7234.
32. Welikala R, Foster P, Whincup P, Rudnicka AR, Owen CG, Strachan D, Barman S: **Automated arteriole and venule classification using deep learning for retinal images from the UK Biobank cohort**. *Computers in biology and medicine* 2017, **90**:23-32.
33. Tapp RJ, Owen CG, Barman SA, Welikala RA, Foster PJ, Whincup PH, Strachan DP, Rudnicka AR, UK Biobank Eye VC: **Retinal vascular tortuosity and diameter associations with adiposity and components of body composition**. *Obesity* 2020, **28**(9):1750-1760.
34. Tapp RJ, Owen CG, Barman SA, Welikala RA, Foster PJ, Whincup PH, Strachan DP, Rudnicka AR: **Associations of Retinal Microvascular Diameters and Tortuosity With Blood Pressure and Arterial Stiffness: United Kingdom Biobank**. *Hypertension* 2019, **74**(6):1383-1390.
35. Rudnicka AR, Welikala R, Barman S, Foster PJ, Luben R, Hayat S, Khaw KT, Whincup P, Strachan D, Owen CG: **Artificial intelligence-enabled retinal vasculometry for prediction of circulatory mortality, myocardial infarction and stroke**. *Br J Ophthalmol* 2022.
36. McGrory S, Taylor AM, Pellegrini E, Ballerini L, Kirin M, Doubal FN, Wardlaw JM, Doney AS, Dhillon B, Starr JM: **Towards standardization of quantitative retinal vascular parameters: comparison of SIVA and VAMPIRE measurements in the Lothian Birth Cohort 1936**. *Translational vision science & technology* 2018, **7**(2):12-12.
37. Perez-Rovira A, MacGillivray T, Trucco E, Chin K, Zutis K, Lupascu C, Tegolo D, Giachetti A, Wilson PJ, Doney A: **VAMPIRE: vessel assessment and measurement platform for images of the REtina**. In: *2011 Annual International Conference of the IEEE Engineering in Medicine and Biology Society: 2011*: IEEE; 2011: 3391-3394.
38. Remond P, Aptel F, Cunnac P, Labarere J, Palombi K, Pepin J-L, Pollet-Villard F, Hogg S, Wang R, MacGillivray T: **Retinal vessel phenotype in patients with nonarteritic anterior ischemic optic neuropathy**. *American journal of ophthalmology* 2019, **208**:178-184.
39. Azanan MS, Chandrasekaran S, Rosli ES, Chua LL, Oh L, Chin TF, Yap TY, Rajagopal R, Rajasuriar R, MacGillivray T: **Retinal Vessel Analysis as a Novel Screening Tool to Identify Childhood Acute Lymphoblastic Leukemia Survivors at Risk of Cardiovascular Disease**. *Journal of pediatric hematology/oncology* 2020, **42**(6):e394-e400.
40. Ko F, Muthy ZA, Gallacher J, Sudlow C, Rees G, Yang Q, Keane PA, Petzold A, Khaw PT, Reisman C: **Association of retinal nerve fiber layer thinning with current and future cognitive decline: a study using optical coherence tomography**. *JAMA neurology* 2018, **75**(10):1198-1205.

For peer review only

41. Cornelis MC, Wang Y, Holland T, Agarwal P, Weintraub S, Morris MC: **Age and cognitive decline in the UK Biobank**. *PloS one* 2019, **14**(3):e0213948.
42. Chua SY, Lascaratos G, Atan D, Zhang B, Reisman C, Khaw PT, Smith SM, Matthews PM, Petzold A, Strouthidis NG: **Relationships between retinal layer thickness and brain volumes in the UK Biobank cohort**. *European Journal of Neurology* 2021, **28**(5):1490-1498.
43. Lyall DM, Cullen B, Allerhand M, Smith DJ, Mackay D, Evans J, Anderson J, Fawns-Ritchie C, McIntosh AM, Deary IJ: **Cognitive test scores in UK Biobank: data reduction in 480,416 participants and longitudinal stability in 20,346 participants**. *PloS one* 2016, **11**(4):e0154222.
44. Riley RD, Snell KI, Ensor J, Burke DL, Harrell Jr FE, Moons KG, Collins GS: **Minimum sample size for developing a multivariable prediction model: PART II-binary and time-to-event outcomes**. *Statistics in medicine* 2019, **38**(7):1276-1296.
45. Petermann-Rocha F, Lyall DM, Gray SR, Esteban-Cornejo I, Quinn TJ, Ho FK, Pell JP, Celis-Morales C: **Associations between physical frailty and dementia incidence: a prospective study from UK Biobank**. *The Lancet Healthy Longevity* 2020, **1**(2):e58-e68.
46. Standard B: **Test charts for determining distance visual acuity: BS 4274-1968**. *British Standards Institute* 1968.
47. Tikkanen E, Gustafsson S, Ingelsson E: **Associations of fitness, physical activity, strength, and genetic risk with cardiovascular disease: longitudinal analyses in the UK Biobank Study**. *Circulation* 2018, **137**(24):2583-2591.
48. Perez-Cornago A, Key TJ, Allen NE, Fensom GK, Bradbury KE, Martin RM, Travis RC: **Prospective investigation of risk factors for prostate cancer in the UK Biobank cohort study**. *British journal of cancer* 2017, **117**(10):1562-1571.
49. Allen NE, Sudlow C, Peakman T, Collins R, biobank U: **UK biobank data: come and get it**. In., vol. 6: American Association for the Advancement of Science; 2014: 224ed224-224ed224.
50. Ntuk UE, Gill JM, Mackay DF, Sattar N, Pell JP: **Ethnic-specific obesity cutoffs for diabetes risk: cross-sectional study of 490,288 UK biobank participants**. *Diabetes care* 2014, **37**(9):2500-2507.
51. Millett ER, Peters SA, Woodward M: **Sex differences in risk factors for myocardial infarction: cohort study of UK Biobank participants**. *bmj* 2018, **363**.
52. Gallacher KI, McQueenie R, Nicholl B, Jani BD, Lee D, Mair FS: **Risk factors and mortality associated with multimorbidity in people with stroke or transient ischaemic attack: a study of 8,751 UK Biobank participants**. *Journal of comorbidity* 2018, **8**(1):1-8.
53. Ma H, Li X, Sun D, Zhou T, Ley SH, Gustat J, Heianza Y, Qi L: **Association of habitual glucosamine use with risk of cardiovascular disease: prospective study in UK Biobank**. *bmj* 2019, **365**.
54. Gao L, Li P, Cui L, Wong PM, Johnson-Akeju O, Lane J, Saxena R, Scheer F, Hu K: **Sleep disturbance and incident Alzheimer's disease: A UK Biobank study of 502,538 middle-aged to older participants: Biomarkers (non-neuroimaging): Alzheimer's disease incidence, risk factors and biomarkers**. *Alzheimer's & Dementia* 2020, **16**:e044575.
55. Veronese N, Yang L, Piccio L, Smith L, Firth J, Marx W, Giannelli G, Caruso MG, Cisternino AM, Notarnicola M: **Adherence to a healthy lifestyle and multiple**

For peer review only

sclerosis: a case–control study from the UK Biobank. *Nutritional neuroscience* 2020:1-9.
56. Petzold A, Chua SY, Khawaja AP, Keane PA, Khaw PT, Reisman C, Dhillon B, Strouthidis NG, Foster PJ, Patel PJ: **Retinal asymmetry in multiple sclerosis**. *Brain* 2021, **144**(1):224-235.
57. Petzold A, Fraser CL, Abegg M, Alroughani R, Alshowaeir D, Alvarenga R, Andris C, Asgari N, Barnett Y, Battistella R: **Diagnosis and classification of optic neuritis**. *The Lancet Neurology* 2022.
58. Cumberland PM, Rahi JS: **Visual function, social position, and health and life chances: the UK biobank study**. *JAMA ophthalmology* 2016, **134**(9):959-966.
59. Chan MP, Grossi CM, Khawaja AP, Yip JL, Khaw K-T, Patel PJ, Khaw PT, Morgan JE, Vernon SA, Foster PJ: **Associations with intraocular pressure in a large cohort: results from the UK Biobank**. *Ophthalmology* 2016, **123**(4):771-782.
60. Shah RL, Guggenheim JA: **Genome-wide association studies for corneal and refractive astigmatism in UK Biobank demonstrate a shared role for myopia susceptibility loci**. *Human genetics* 2018, **137**(11):881-896.
61. Wood A, Guggenheim JA: **Refractive error has minimal influence on the risk of age-related macular degeneration: a Mendelian randomization study**. *American journal of ophthalmology* 2019, **206**:87-93.
62. Craig JE, Han X, Qassim A, Hassall M, Cooke Bailey JN, Kinzy TG, Khawaja AP, An J, Marshall H, Gharahkhani P: **Multitrait analysis of glaucoma identifies new risk loci and enables polygenic prediction of disease susceptibility and progression**. *Nature genetics* 2020, **52**(2):160-166.
63. de Jong FJ, Schrijvers EM, Ikram MK, Koudstaal PJ, de Jong PT, Hofman A, Vingerling JR, Breteler MM: **Retinal vascular caliber and risk of dementia: the Rotterdam study**. *Neurology* 2011, **76**(9):816-821.
64. Mutlu U, Cremers LG, De Groot M, Hofman A, Niessen WJ, Van Der Lugt A, Klaver CC, Ikram MA, Vernooij MW, Ikram MK: **Retinal microvasculature and white matter microstructure: the Rotterdam Study**. *Neurology* 2016, **87**(10):1003-1010.
65. Mutlu U, Bonnemaijer PW, Ikram MA, Colijn JM, Cremers LG, Buitendijk GH, Vingerling JR, Niessen WJ, Vernooij MW, Klaver CC: **Retinal neurodegeneration and brain MRI markers: the Rotterdam Study**. *Neurobiology of aging* 2017, **60**:183-191.
66. Sudlow C, Gallacher J, Allen N, Beral V, Burton P, Danesh J, Downey P, Elliott P, Green J, Landray M: **UK biobank: an open access resource for identifying the causes of a wide range of complex diseases of middle and old age**. *PLoS medicine* 2015, **12**(3):e1001779.
67. Biobank U: **UK Biobank ethics and governance framework**. In.; 2015.

For peer review only

BMJ Open

Cohort Profile: Rationale and Methods of UK Biobank Repeat Imaging Study Eye Measures to Study Dementia

Journal:	*BMJ Open*
Manuscript ID	bmjopen-2022-069258.R1
Article Type:	Cohort profile
Date Submitted by the Author:	17-May-2023

Complete List of Authors:	Foster, Paul; NIHR Moorfields Biomedical Research Centre, Moorfields Eye Hospital NHS Foundation Trust<br>Atan, Denize; University of Bristol<br>Khawaja, Anthony; 1. NIHR Biomedical Research Centre at Moorfields Eye Hospital NHS Foundation Trust & UCL Institute of Ophthalmology, London, UK<br>Lotery, Andrew; University of Southampton<br>MacGillivray, Tom; Queens Medical Research Institution, Clinical Research Imaging Centre<br>Owen, Christopher; St George's, University London, Population Health Research Institute<br>Patel, Praveen; Moorfields Eye Hospital NHS Foundation Trust, NIHR Biomedical Research Centre<br>Petzold, Axel; Queen Square Institute of Neurology, UCL, Department of Molecular Neurosciences, Moorfields Eye Hospital and The National Hospital for Neurology and Neurosurgery; Amsterdam UMC, Departments of Neurology, Ophthalmology and Expertise Center for Neuro-ophthalmology<br>Rudnicka, Alicja; SGUL University of London<br>Sun, Zihan; University College London, Institute of Ophthalmology<br>Sheard, Simon; UK Biobank<br>Allen, Naomi; University of Oxford, Nuffield Department of Population Health; UK Biobank

**Primary Subject Heading**:	Epidemiology
Secondary Subject Heading:	Ophthalmology
Keywords:	OPHTHALMOLOGY, EPIDEMIOLOGY, Dementia < NEUROLOGY

SCHOLARONE™
Manuscripts

I, the Submitting Author has the right to grant and does grant on behalf of all authors of the Work (as defined in the below author licence), an exclusive licence and/or a non-exclusive licence for contributions from authors who are: i) UK Crown employees; ii) where BMJ has agreed a CC-BY licence shall apply, and/or iii) in accordance with the terms applicable for US Federal Government officers or employees acting as part of their official duties; on a worldwide, perpetual, irrevocable, royalty-free basis to BMJ Publishing Group Ltd ("BMJ") its licensees and where the relevant Journal is co-owned by BMJ to the co-owners of the Journal, to publish the Work in this journal and any other BMJ products and to exploit all rights, as set out in our licence.

The Submitting Author accepts and understands that any supply made under these terms is made by BMJ to the Submitting Author unless you are acting as an employee on behalf of your employer or a postgraduate student of an affiliated institution which is paying any applicable article publishing charge ("APC") for Open Access articles. Where the Submitting Author wishes to make the Work available on an Open Access basis (and intends to pay the relevant APC), the terms of reuse of such Open Access shall be governed by a Creative Commons licence – details of these licences and which Creative Commons licence will apply to this Work are set out in our licence referred to above.

Other than as permitted in any relevant BMJ Author's Self Archiving Policies, I confirm this Work has not been accepted for publication elsewhere, is not being considered for publication elsewhere and does not duplicate material already published. I confirm all authors consent to publication of this Work and authorise the granting of this licence.

For peer review only

Cohort Profile: Rationale and Methods of UK Biobank Repeat Imaging Study Eye Measures to Study Dementia

Authors:
Paul J Foster[1], Denize Atan[2,3], Anthony P. Khawaja[1], Andrew J. Lotery[4], Tom MacGillivray[5], Christopher G Owen[6], Praveen J. Patel[1], Axel Petzold[7,8], Alicja R. Rudnicka[6], Zihan Sun[1], Simon Sheard[9], Naomi Allen[9,10], *On behalf of UK Biobank and UK Biobank Eye and Vision Consortium*

Affiliations:
1. NIHR Biomedical Research Centre at Moorfields Eye Hospital NHS Foundation Trust and UCL Institute of Ophthalmology, London, UK
2. Bristol Eye Hospital, University Hospitals Bristol & Weston NHS Foundation Trust, Bristol, UK
3. Translational Health Sciences, Bristol Medical School, University of Bristol, Bristol, UK
4. Clinical Neurosciences Group, Clinical and Experimental Sciences, Faculty of Medicine, University of Southampton, Southampton, UK
5. Centre for Clinical Brain Sciences, University of Edinburgh, Edinburgh, UK
6. Population Health Research Institute, St George's, University of London, London, UK
7. Queen Square Institute of Neurology, UCL, Department of Molecular Neurosciences, Moorfields Eye Hospital and The National Hospital for Neurology and Neurosurgery, London, UK
8. Departments of Neurology, Ophthalmology and Expertise Center for Neuro-ophthalmology, Amsterdam UMC, Amsterdam, Netherlands
9. UK Biobank, Stockport, UK
10. Nuffield Department of Population Health, University of Oxford, Oxford, UK

Correspondence to:
Prof Paul J Foster
Address: UCL Institute of Ophthalmology, 11-43 Bath Street, London EC1V 9EL
Email address : p.foster@ucl.ac.uk

For peer review only

ABSTRACT

Purpose: The retina provides biomarkers of neuronal and vascular health that offer promising insights into cognitive ageing, mild cognitive impairment (MCI) and dementia. This article described the rationale and methodology of eye and vision assessments with the aim of supporting the study of dementia in the UK Biobank Repeat Imaging study.

Participants: UK Biobank is a large-scale, multicentre, prospective cohort containing in-depth genetic, lifestyle, environmental and health information from half a million participants aged 40-69 enrolled in 2006-2010 across the UK. A subset (up to 60,000 participants) of the cohort will be invited to the UK Biobank Repeat Imaging Study to collect repeated brain, cardiac and abdominal magnetic resonance imaging (MRI) scans, whole-body DEXA (Dual-energy x-ray absorptiometry), carotid ultrasound, as well as retinal optical coherence tomography (OCT) and colour fundus photographs.

Findings to date: UK Biobank has helped make significant advances in understanding risk factors for many common diseases, including for dementia and cognitive decline. Ophthalmic genetic and epidemiology studies have also benefited from the unparalleled combination of very large numbers of participants, deep phenotyping, and longitudinal follow-up of the cohort, with comprehensive health data linkage to disease outcomes. In addition, we have used UK Biobank data to describe the relationship between retinal structures, cognitive function and brain MRI-derived phenotypes.

Future plans: The collection of eye-related data (e.g., OCT), as part of the UK Biobank Repeat Imaging study, will take place in 2022-2028. The depth and breadth and longitudinal nature of this dataset, coupled with its open-access policy, will create a major new resource for dementia diagnostic discovery and to better understand its association with co-morbid diseases. Additionally, the broad and diverse data available in this study will support research into ophthalmic diseases and various other health outcomes beyond dementia.

For peer review only - http://bmjopen.bmj.com/site/about/guidelines.xhtml

STRENGTHS AND LIMITATIONS OF THIS STUDY

Strengths

- World's largest prospective, longitudinal multi-modal imaging cohort with unprecedented power for analysis of determinants of a wide range of health outcomes.
- Exceptional added value from the size, depth, and quality of the cross-sectional and longitudinal MRI data on the eye, brain, body and imaging of heart, carotids, together with linkage to electronic health records, through which overt dementia and Alzheimer's disease can be identified.
- Optimal timing to study cognitive impairment (age distribution: ~80% ≥ 60 years and ~65% ≥ 65 years)

Limitations

- ○ Consistency of measurements between imaging devices over time, particularly with use of different OCT devices.
- ○ Healthier participants compared to the general population.

For peer review only

INTRODUCTION

Dementia refers to a heterogeneous group of neurodegenerative disorders affecting 46.8 million people globally.[1-3] Alzheimer's disease (AD) is the commonest, affecting 60-80% of people with dementia.[4, 5] Usually, a long prodromal period of up to 20 years of progressive cerebral atrophy is detectable on magnetic resonance imaging (MRI) scans and using body fluid biomarkers for neurodegeneration before AD is diagnosed.[2] These observations lead to a biological, rather than a clinical definition of AD.[6] To date, the majority of candidate drugs for slowing cognitive decline in AD or other dementias have failed in clinical trials[7], probably because they are used too late in the natural history when irreversible, advanced degeneration has already set in.[8, 9] Global rollout of screening and disease progression monitoring strategies for AD based on MRI scans is precluded by their high cost and frequently limited availability. Body fluid biomarkers might provide ways of stratifying or diagnosing dementias but will remain complimentary to structural imaging biomarkers because of their lack of diagnostic specificity and are not recommended as a screening test.[10]

The eye provides insights into the risk or presence of some major systemic diseases, including hypertension and diabetes, as well as changes associated with cognitive ageing and neurodegeneration.[11-13] As an alternative to MRI or plasma biomarkers, optical coherence tomography (OCT) offers a rapid, low-cost and non-invasive method for obtaining high-resolution (3-5μm) images of the retina at the back of the eye – the only part of the central nervous system (CNS) that can be visualized directly. The laminated structure of the retina enables the direct monitoring of neurodegeneration at a near cellular level *in vivo* at a resolution that is more detailed than for any other non-invasive, in vivo imaging modalities. There is strong evidence that quantitative OCT measurements are associated with concurrent cognitive impairment and future cognitive decline and dementia.[14, 15] Additionally, OCT

For peer review only - http://bmjopen.bmj.com/site/about/guidelines.xhtml

methods may directly monitor related vascular pathology: amyloid microangiopathy affects retinal and choroidal vasculature, as well as that in the cerebrum with AD.[16] Thus, retinal OCT scans offer the means to identify individuals at high risk of developing AD, providing them with opportunities to change their lifestyles or enter drug trials to delay or avert the onset of dementia. OCT scans are also a sensitive way to monitor patients for neurotoxic side-effects of novel drug treatments. The ability to directly measure specific neuronal layers and microvascular characteristics in detail may provide a surrogate outcome marker for the CNS more generally and potentially enhance the power to detect disease much earlier than methods based on clinical history and genetic factors.

Several genetic (e.g. *APOE*), comorbid (e.g. diabetes, hypertension, depression, obesity), and lifestyle factors (e.g. low educational attainment and smoking) have been associated with increased AD risk.[17-19] However, observational epidemiological studies cannot distinguish cause from effect and are vulnerable to bias from reverse causation and confounders. The analyses by Norton and Larsson illustrate how different disease risk factors can co-exist and are often correlated, but they do not independently increase the risk of AD.[19, 20] It also highlights the importance of identifying causal risk factors for dementia in designing upstream public health policies and social policies to reduce disease risk, clinical trials for new AD drugs and basic science research to understand the underlying mechanisms of dementia development. The large size of the cohort and the associated healthcare, imaging and genetic data make UK Biobank uniquely valuable for disambiguation of associations from causal comorbidities both for patient stratification and for elucidation of underlying mechanisms.

The research gaps, as mentioned earlier, motivated us to develop a major new resource for dementia diagnostic discovery and to better understand the association with co-morbid diseases by adding rapid, low-cost OCT to the anticipated UK Biobank Repeat

For peer review only

Imaging study, alongside ancillary testing of autorefraction/keratometry and fragmented letter test (FLT).[21] The objectives of this article are to describe (1) the process of test selection (2) the methodology for eye and vision measures in the UK Biobank Repeat Imaging study; (3) the baseline characteristics of the study population in this study.

COHORT DESCRIPTION

UK Biobank

UK Biobank is a large-scale biomedical database and research resource containing in-depth genetic and health information from over 500,000 participants aged 40-69 enrolled across the UK between 2006 and 2010. Detailed study protocols are available on the UK Biobank website (https://www.ukbiobank.ac.uk/). It has become the pre-eminent biomedical research platform for studying the aetiology of common diseases of later life. During the baseline assessment, extensive sociodemographic, lifestyle, and health-related information was collected through a touch screen questionnaire and oral interview, and a wide range of physical measurements was performed.[22, 23] Participants also provided biological samples for genotyping, haematological, biochemistry, metabolomics and proteomics assays for the full cohort.[24]

UK Biobank received approval from the National Information Governance Board for Health and Social Care and the National Health Service Northwest Centre for Research Ethics Committee (Ref: 11/NW/0382). UK Biobank is compliant with the previous Data Protection Act and the more recent General Data Protection Regulation (GDPR) implemented in 2018. For the GDPR, participants were contacted by email or post to explain how UK Biobank meets the requirements of the new regulations (https://www.ukbiobank.ac.uk/gdpr/). At the baseline visit, ophthalmic assessments were performed on a subset of participants between 2009-2010 at 6 of 22 UK Biobank assessment centres, including visual acuity, autorefraction, keratometry, intraocular pressure, corneal biomechanics, and retinal imaging

For peer review only - http://bmjopen.bmj.com/site/about/guidelines.xhtml

comprising disc/macular digital colour photographs and a 3D macular OCT. Over 110,000 participants have completed the visual acuity, refractive error, and intraocular pressure measurements; and ~67,000 participants underwent retinal imaging. Detailed information on the baseline eye and vision measures has been published elsewhere [22].

The Repeat Imaging Sub-study

In 2014, UK Biobank launched the world's largest multimodal imaging study, intending to include baseline magnetic resonance imaging (MRI) of the brain, heart and abdomen, whole-body DEXA (Dual-energy x-ray absorptiometry) and carotid Doppler ultrasound on up to 100,000 participants. Detailed methods of the UK Biobank imaging enhancement were published elsewhere.[25] Although imaging 100,000 participants is a unique and powerful enhancement to the UKB resource, many valuable insights could only be gained from observing the change in imaging phenotypes over time. Recognising the importance of serial measurements, up to 60,000 of those in the imaging enhancement study will be invited to undergo repeat multimodal imaging between 2019-2028. As part of the repeat imaging study, data collection of the eye measures (e.g., OCT) is anticipated to take place from 2022-2028. The specific study design is as follows:

- All UK Biobank participants who have previously attended a baseline brain and body imaging visit will be invited to attend a repeat imaging visit (the invitation will specify the same imaging centre as their baseline imaging visit to minimize measurement error caused by differences between scanners at different centres).
- Appointment slots are planned in groups of 3 to minimize equipment downtime and to maximize participant throughput and data quality.
- On arrival, those who accept will be asked to consent to the study, and each participant will then undergo a pre-screening safety assessment.
- The 3 participants would then progress to the imaging modalities as follows:

For peer review only

   - Participant #1 => Brain MRI
   - Participant #2 => Abdomen and heart MRI
   - Participant #3 => DeXA, ultrasound and OCT
- Each imaging modality "group" takes approximately 40 minutes, after which the participants will move to the next modality, then switch again after 40 minutes so that each member of the group of 3 has visited all three imaging measurement stations over a 2-hour period.
- The participants then all progress to the non-imaging parts of the visit where they will complete questionnaires, have physical measures, and give biological samples, which mirrors much of the initial (2006-2010) baseline visit.
- As one group of 3 participants exits the imaging part of the visit, the next group of 3 are ready to enter, thus ensuring that the imaging part of the visit is fully utilized.
- This process will repeat for five groups of 3 people (15 total) on 7 days per week at each of UK Biobank's 4 dedicated imaging centres.

Study location

This multisite study will be run from four dedicated UK Biobank Imaging Centres across the UK (Newcastle upon Tyne, Stockport, Reading and Bristol). These 4 centres help ensure most participants are within a reasonable distance to attend a scanning visit. As far as is reasonably practical, maintaining the same instruments and software/firmware across the sites and all phases of the UK Biobank project will ensure consistency and comparability of results from the start of the baseline imaging project to the end of the repeat imaging program. UK Biobank built the following strategies to reduce variability across the different sites:

For peer review only - http://bmjopen.bmj.com/site/about/guidelines.xhtml

- The sites are each populated with the same equipment (same manufacturer, same model, same software/firmware etc.) configured with the same protocols and the same settings.
- MR scanner settings/performance across all four sites is monitored by UK Biobank's full-time in-house MR physicist with continuous quality assurance processes to identify and resolve quality issues that may arise.
- All staff are trained to standard operating procedures, and (in the case of the imaging element) compliance/consistency is overseen by an in-house senior radiographer and an in-house MR physicist. The non-imaging aspects are overseen by UK Biobank's dedicated "Training and Monitoring" team.
- Systems are already in place to ensure appropriate levels of training for all operational staff, monitored via Clinic Training Assessments/Training Matrices. These will be extended to cover the OCT measures: appropriate training will be provided, training assessments/matrices extended to cover these measures, and performance monitored.
- Imaging data are routinely made available to members of the project's expert working group, which is made up of experts in each of the imaging modalities; this group monitors the project progress, periodically provides training interventions and critically, periodically/routinely provides an independent view of performance and data output. A similar approach will be taken regarding the OCT measures with data made available to the Eye Consortium members listed in this application for quality control purposes.

Recruitment

The UK Biobank cohort includes a committed and engaged group of participants who are regularly invited for follow up activities: the typical response rate to online surveys is >50%, and there have been very few withdrawals from the study since recruitment

For peer review only - http://bmjopen.bmj.com/site/about/guidelines.xhtml

(<0.2%). Regular communications with the cohort (via newsletters, participant meetings, study update meetings and the participant section of the UK Biobank website: www.ukbiobank.ac.uk/explore-your-participation) help to maintain enthusiasm for and engagement with the study. Direct telephone communication with individual participants regarding new sub-studies or general participation questions via a dedicated "Participant Contact Centre" (PCC) provides personalized information and reassurance.

This study will use the same invitation protocol as the UK Biobank imaging enhancement study (2014-2023)[25]. The planned protocol for this repeat imaging study involves:

- E-mail/postal explanation of the study and invitation to book an appointment.
- A telephone call to book an appointment and perform safety pre-screening via Participant Resource Centre (PRC).
- Assessment at the nearest of four imaging centres across the UK (Stockport, Newcastle, Reading and Bristol) to minimize travel time and maximize participant attendance.

At the start of the pandemic (lockdown in the UK in March 2020), 50,000 of the target 100,000 participants had been imaged. Participant questionnaires on completion of baseline imaging visit indicate >90% would be happy to undertake a repeat imaging visit. Pilot studies involving a few thousand participants have demonstrated ~60% acceptance rates, providing confidence that ~60,000 could be recruited for this repeat imaging study.

Examination procedures

The whole-body imaging modalities have been extensively detailed elsewhere[25]; this article only describes the scope of eye and vision measurements in the Repeat Imaging study. The Topcon Triton OCT platform is being used to obtain OCT images in this study. The Triton platform uses ultra-highspeed swept-source (SS) OCT technology with a central wavelength of 1050 microns that penetrates deeper than the retina, allowing visualization of

For peer review only - http://bmjopen.bmj.com/site/about/guidelines.xhtml

the choroid and the vasculature therein.[26] The platform also takes colour retinal fundus photographs immediately after the OCT scans, allowing measurement of the optic disc and retinal vessel metrics (including retinal vessel calibre and tortuosity). The Topcon Triton supports wide-angle 12 mm x 9 mm scans that include the optic disc and macula in a single scan.

Widefield SS-OCT enables quantitative measurements of several candidate biomarkers, including but not limited to total macular retinal thickness, macular inner retinal sublayer thicknesses, peripapillary retinal nerve fibre layer thickness, choroidal vascularity index, retinal arteriolar and retinal venular calibres, retinal vascular fractal dimension, retinal vascular tortuosity. Details of the candidate biomarkers are summarized in Table 1.

OCT Image Processing

Total retinal thickness and segmented values for retinal sublayer thicknesses for macula and optic nerve scans are generated by the current generation OCT devices, using FDA approved algorithms, during the examination. In contrast to the processing of baseline UK Biobank macular OCT scans[27], they do not generally require the development of new processing pipelines (apart from measures of the choroidal vascular layer, which are now possible thanks to greater depth of imaging than was previously possible with older OCT technology). Fundamental to both the challenge and the opportunity that would be provided by UK Biobank OCT imaging is that modern retinal imaging software can measure changes that would be imperceptible to, or missed by, a human grader. In operations featuring large-scale data collection, a small proportion of the imaging is likely to be insufficient quality for automated analysis. Problems may also arise with image acquisition, for instance, due to some study participants presenting with ocular pathology. Thus, the first step in an analysis pipeline is to assess the image quality and discard images that cannot be adequately measured. Subsequent analysis of vasculometrics from retinal photographs would include

For peer review only

automated vessel segmentation followed by classification of arterioles and venules (see below). For OCT, algorithms delineate the borders of the internal limiting membrane (ILM) and the RNFL to give the measurement of RNFL thickness, a biomarker of axonal loss affecting retinal ganglion cells and the optic nerves. The thickness of the RNFL is evaluated using the standard TSNIT (temporal, superior, nasal, inferior, temporal) mapping that subdivides the measurements and colour codes statistical significance compared with a database of normal healthy values. Further delineation of boundaries enables quantitative mapping of the ganglion cell layer (GCL) and inner plexiform layer (IPL) thicknesses, a marker of neuronal somatic loss [13, 28].

Although the processing of quantitative retinal vasculometric data is not routinely used in clinical settings, we have developed and validated a fully automated AI-enabled retinal image analysis system (QUARTZ) for extracting vessel maps and quantifying retinal vasculometry (including vessel size and tortuosity), which we will use to create the image processing pipeline. The system overcomes many of the difficulties of earlier vasculometry approaches, particularly by being fully automated.[29, 30] QUARTZ has been demonstrated to be highly robust, capable of processing large datasets with automated image quality assessments, resulting in accurate, reliable and high levels of vessel segmentation. To date, QUARTZ has measured approximately 4 million vessel segments from over 190,000 images from 95,000 participants of two very large population-based cohorts (UK Biobank and EPIC-Norfolk). This system has been developed specifically for use on TOPCON macular centred images.

In brief, the QUARTZ system distinguishes between right and left eyes, venules and arterioles (with 87% accuracy using AI-enabled deep learning), identifies vessel segments and centreline coordinates and outputs measures of vessel width and tortuosity (based on the mean change in chord length between successive divisions of the vessel).[31, 32] The system

For peer review only - http://bmjopen.bmj.com/site/about/guidelines.xhtml

298 obtains 10-20 thousands of measurements of width and tortuosity from the whole retinal
299 image (dependent on image quality), not just selected vessels lying within concentric areas
300 centred on the disc. Measures are summarized using mean width and tortuosity, weighted by
301 segment length, for arterioles and venules separately for each image. QUARTZ measures in
302 UK Biobank have previously shown that venular width and tortuosity are associated with
303 markers of adiposity[33] and that both arteriolar and venular width and arteriolar tortuosity
304 show strong inverse associations with blood pressure (systolic and diastolic) and arterial
305 stiffness index.[34] More importantly, prognostic models using QUARTZ vasculometry
306 measures perform very well at predicting circulatory mortality and at least as well as
307 established risk scores in the prediction of stroke and myocardial infarction, remarkably
308 without the need for either a blood test or blood pressure measurement.[35] Given the
309 identification of vessel maps, these could be inputted into other systems (i.e., the VAMPIRE
310 system) with additional vasculometry summaries, such as fractal analyses to quantify the
311 complexity of the arteriolar and venular components of the retinal vascular network.
312 Marrying the automated functionality of QUARTZ with VAMPIRE will afford a more in-
313 depth characterization of the vessel complex on an unprecedented scale.

314 The VAMPIRE (Vascular Assessment and Measurement Platform for Images of the
315 REtina) system is an international collaborative project designed to quantify retinal vascular
316 morphometry with large collections of fundus photographs. The system provides automatic
317 detection of retinal landmarks and quantifies some key parameters used frequently in
318 investigative studies - vessel width, vessel branching coefficients, tortuosity, and fractal
319 analyses. Detailed definitions have been reported elsewhere.[36, 37] In general, it computes
320 149 measurements per image, including basic statistics. Thirty-nine are width-related: central
321 retinal arteriolar equivalent (CRAE), central retinal venular equivalent (CRVE), retinal
322 arteriovenous ratio (AVR), basic statistics (mean, median, standard deviation, maximum,

For peer review only

minimum), width gradients along vessels, average ratio length-diameter at branching points, by arteries and veins; 104 are tortuosity measurements, computed by different algorithms and with the statistics listed above; 6 are fractal dimension coefficients. All measures are calculated by vessel type (arteriole or venule) and region (zone, whole image, quadrants). VAMPIRE is a validated software application and has been extensively used in several international studies.[36, 38, 39]

Patient and public involvement

UK Biobank maintains a website to keep participants and researchers up to date on the study (http://www.ukbiobank.ac.uk/news/). Eye and vision-related publications resulting from UK Biobank are maintained at (https://www.ukbiobankeyeconsortium.org.uk/publications). UK Biobank also holds regular events to inform the participants about the imaging study and the latest research. In addition, UK Biobank has a Twitter feed (@uk_biobank). The study was set up by the Medical Research Council (MRC), Department of Health (DoH), and Wellcome Trust with input from major patient representative organizations. An annual scientific meeting is recorded and available to the public as a webcast.

Statistical Analysis Plan

Baseline ocular characteristics will be summarized as mean (standard deviation) for continuous variables and number (%) for categorical variables.

Primary aims would be to examine:

1) cross-sectional associations between retinal biomarkers, measures of cognitive performance and brain-volume from MRI imaging
2) the comparative performance of retinal biomarkers for risk stratification, to identify those with cognitive impairment.

For peer review only - http://bmjopen.bmj.com/site/about/guidelines.xhtml

3) the comparative performance of retinal biomarkers to detect those with longitudinal decline in cognitive performance.

Previous work within UK Biobank examining RNFL measures in relation to mild cognitive impairment, showed that those in the lowest quintile of RNFL thickness were 11% (95% CI: 2% to 21%) more likely to fail on at least one of four cognitive tests.[40] This shows that RNFL measures have the potential to identify those at higher risk of cognitive impairment. After vigorous image quality control, the proposed imaging of a further 60,000 participants will provide 45,000-55,000 participants with good-quality retinal images for quantification of individual components of the RNFL and potential to extract detailed retinal vasculometric measures. This large sample size, will have 99% power (alpha = 0.001) to detect at least 0.03 standard deviation change in the cognitive score[41] or brain measures[42] (based on F-tests of linear regression coefficients from cross-sectional analyses) per 1 standard deviation increase in any retinal biomarker (RNFL or retinal vasculometric measure). Cross-sectional analyses using multiple linear regression will quantify the dose response relationship between cognitive score with considerable power to evaluate in the region of 30 candidate predictors (retinal biomarkers, age, sex, geographical location, height, refraction, intraocular pressure, smoking status, socioeconomic positions and established cardiovascular risk markers).[43] This will allow the independent contribution of retinal biomarkers as a predictor of cognitive performance to be realized with considerable precision,[44] across a spectrum of cognitive scores.[43]

Given that UK Biobank has longitudinal data on cognitive change (with repeated measures available from online questionnaires and performed in-person at the imaging assessments), the study would be uniquely placed to assess the determinants of cognitive decline in middle-later life. For prospective evaluation the rates of dementia would also be pivotal in relation to prior cognitive performance. In UK Biobank, the annual incidence of

For peer review only

dementia among those aged ≥ 60 years old is approximately 2.5 per 1000 person years.[45] Therefore, within 2 years of retinal image capture there would be approximately 250 cases of dementia per 45,000-55,000 participants. The longitudinal nature of the data will allow models to be developed for incident cognitive outcomes / neurodegenerative events using multivariable Cox proportional hazards models with relevant eye measures (i.e., OCT, retinal vasculometry derived measures) as continuous predictors both with and without inclusion of other parameters, including age at cognitive decline / neurodegenerative onset, sex, ethnicity (although the cohort is largely of white European ancestry), smoking status (current, former and never), alcohol consumption, body mass index, blood pressure, blood biochemistry measures, social deprivation (by postcode), physical activity / sedentary behaviour, and relevant family history where available.

Existing Data

Once recruitment was fully under way, additional measures were incorporated into the baseline assessment, including hearing and arterial stiffness tests, a cardiorespiratory fitness test, and various eye and vision measures, including visual acuity on a computerised system designed to observe logarithm of the minimum angle of resolution (logMAR) principles, and following the British Standard (BS-1968),[46] autorefraction and keratometry, intraocular pressure and corneal biomechanics, and retinal imaging comprising disc/macular digital colour photographs and a 3D macular OCT.[22] After the baseline visit, subsets of participants have supported additional data collection through various enhancements to the study. These have included: a complete repeat of the baseline assessment, collection of physical activity data over 7-days by wearing accelerometers, and regular online questionnaires covering various topics such as diet, cognitive function, occupational history, mental wellbeing, gastrointestinal health and pain. All participants provided consent for their health to be followed-up through linkage to health-related records, which currently includes

For peer review only

death, cancer, and hospital inpatient records for the entire cohort. Although UK Biobank is not representative of the entire UK population, the large sample size and variation across all levels of measures nonetheless enable a valid assessment of many exposure-outcome relationships to be made. All publications using UK Biobank data are available on the website (https://www.ukbiobank.ac.uk/enable-your-research/publications). Eye and vision-related publications resulting from UK Biobank is maintained at (https://www.ukbiobankeyeconsortium.org.uk/publications).

In brief, based on data from UK Biobank participants attending the baseline imaging assessment to date (N=48,998), the mean (standard deviation) age was 55.2 (7.6) years; 52% (N=25,290) of them were female. A subset of 13,732 (28%) participants had undergone retinal imaging. As there is a policy for the UK Biobank Repeat Imaging Study to over-sample participants with baseline retinal imaging, the estimated numbers of participants with overlapping retinal imaging and whole-body imaging data in the repeat imaging visit will be more than 16,800. Detailed cognitive scores, APOE genotypes, self-reported comorbidities and medication use are provided in Table 2. In addition to imaging, UK Biobank has implemented a wide range of cognitive function tests since baseline that are relevant to assessing various aspects of cognitive decline and dementia and will be conducted at the repeat imaging and proposed OCT visit (Table 3).

FINDINGS TO DATE

UK Biobank has helped make significant advances in the understanding of risk factors for diseases including cardiovascular diseases, cancer, diabetes, stroke, multiple sclerosis, optic neuritis and dementia.[23, 47-57] Ophthalmic genetics and epidemiology have benefited from the unparalleled combination of very large numbers of participants, very extensive and detailed phenotyping and longitudinal follow-up.[30, 58-62]

For peer review only

In addition, we have used UK Biobank data to describe the relationship between retinal structures and both cognitive function and brain MR image-derived phenotypes.[40, 42] For example, previous work examining RNFL measures in relation to mild cognitive impairment, showed that those in the lowest quintile of RNFL thickness were 11% (95% CI 2.0% to 2.1%) more likely to fail on at least one of four cognitive tests.[40] This indicates that RNFL thickness measurements have the potential to identify those at higher risk of cognitive impairment. Chua *et al* [42] reported that markers of retinal neurodegeneration are associated with smaller brain volumes – macular ganglion cell-inner plexiform layer (GCIPL) thickness, ganglion cell complex (GCC) thickness and total macular thickness were significantly associated with smaller total brain ($p < 0.001$), grey matter and white matter volume ($p < 0.01$), and grey matter volume in the occipital pole ($p < 0.05$); thinner macular GCC and total macular thicknesses were associated with smaller hippocampal volume ($p < 0.02$).

In the context of these results, and the findings of other studies (e.g. The Rotterdam Study),[63-65] we proposed supplementing the testing menu in the UK Biobank Whole Body Repeat Imaging Study with measures that support the discovery and quantification of eye and vision variables that are associated with cognitive ageing and decline, and overt dementia.

COLLABORATION

UK Biobank aims to provide open access data for healthcare-related research. The data are available to all bona fide researchers from the academic, charity, public and commercial sectors in the UK and internationally, without preferential or exclusive access for any user.[66] All interested researchers may apply to access the data via an online application. Strict guidelines are in place to help ensure anonymity and confidentiality of participants' data and samples.[67] We have formed the UK Biobank Eye and Vision Consortium, an 80 person strong group of researchers with interest and expertise in

For peer review only

ophthalmic epidemiology, visual system neurology, and the epidemiology of related diseases such as diabetes and cardiovascular disease (https://www.ukbiobankeyeconsortium.org.uk/).

FURTHER DETAILS

Acknowledgements:

In addition to the listed authors, Prof Rory Collins, Prof Paul Matthews and Dr Mark Effingham participated in scientific discussions which moulded the project that we have outlined here. A funding proposal was developed following discussions with members of the Alzheimer's Drug Discovery Foundation. Meanwhile, we would like to thank all the participants of UK Biobank for their vital contribution to the resource.

Data sharing statement:

This research used data from the UK Biobank Resource, under data access request number 2112.

Contributorship statement:

PJF, APK, PJP & ZS had full access to all the data in the study and take responsibility for the integrity and accuracy of the data analysis. Concept and design: PJF, DA, APK, AJL, TM, CGO, PJP, AP, ARR. Data acquisition, analysis, or interpretation: UK Biobank obtained the data. APK performed data analysis. All authors interpreted data. Critical revision of the manuscript for important intellectual content: all authors. Obtained funding: NA, SS, UK Biobank. All authors approved the final manuscript.

Funding declaration:

The study sponsor/funder was not involved in the design of the study; the collection, analysis, and interpretation of data; writing the report; and did not impose any restrictions regarding the publication of the report. UK Biobank is funded by the Medical Research Council, Wellcome Trust, Department of Health, Scottish Government, the Welsh Assembly Government, British Heart Foundation, Cancer Research UK, NIHR and the Northwest

For peer review only

Regional Development Agency. The UK Biobank Eye and Vision Consortium is supported by grants from Moorfields Eye Charity, the NIHR Biomedical Research Centre at Moorfields Eye Hospital NHS Foundation Trust and UCL Institute of Ophthalmology, the Alcon Research Institute and the International Glaucoma Association (UK).

APK, AP, ZS, PJF and PJP receive salary support from the NIHR BRC at Moorfields Eye Hospital & UCL Institute of Ophthalmology. NA receives salary support from University of Oxford and UK Biobank. PJF receives support from the Desmond Foundation, London, UK. APK is supported by a UKRI Future Leaders Fellowship and an Alcon Research Institute Young Investigator Award. TM acknowledges support from NHS Lothian R&D and the Clinical Research Facility at the University of Edinburgh.

The authors acknowledge a proportion of our financial support from the UK Department of Health through an award made by the National Institute for Health Research to Moorfields Eye Hospital NHS Foundation Trust and UCL Institute of Ophthalmology for a Biomedical Research Centre for Ophthalmology.

Financial disclosures:

PJF reports personal fees from Allergan, Carl Zeiss, Google/DeepMind and Santen, a grant from Alcon, outside the submitted work. PJP reports grants from Topcon Inc, outside the scope of the current report. APK reports personal fees from Abbvie, Aerie, Google Health, Novartis, Reichert, Santen, and Thea, outside the submitted work. AP reports grant support for remyelination trials in multiple sclerosis to the Amsterdam University Medicam Centre, Department of Neurology, MS Centre (RESTORE trial) and UCL, London RECOVER trial; Fight for Sight (nimodipine in optic neuritis trial); royalties or licenses from Up-to-Date (Wolters Kluver) on a book chapter; speaker fees for the Heidelberg Academy; participation on Advisory Board SC Zeiss OCTA Angi-Network, SC Novartis OCTiMS study; equipment: OCTA from Zeiss (Plex Elite).

For peer review only

495

For peer review only

For peer review only - http://bmjopen.bmj.com/site/about/guidelines.xhtml

Table 1. Description of the candidate biomarkers

Biomarkers	View	Description
Total macular retinal thickness	cross-section	distance between the inner boundary of ILM to the lower boundary to RPE
Macular inner retinal sublayer thicknesses		
RNFL thickness	cross-section	distance between ILM to the outer boundary of RNFL
GC-IPL thickness	cross-section	distance between the inner boundary of GCL to the outer boundary of IPL
GCC thickness	cross-section	GC-IPL+ RNFL
Peripapillary RNFL thickness	cross-section	distance between ILM to the outer boundary of RNFL
Choroidal vascularity index	cross-section/*en face*	ratio of vascular luminal area to the total choroidal area
Retinal arteriolar calibres	*en face*	evaluates generalized arteriolar narrowing
Retinal venular calibres	*en face*	evaluates generalized arteriolar narrowing
Retinal vascular fractal dimension	*en face*	measure the vascular pattern complexity
Retinal vascular tortuosity	*en face*	characterized by an abnormal curvature of the vessels, evincing a non-smooth appearance, presenting turns and twists throughout their course.

RNFL=retinal nerve fibre layer; GCIPL=ganglion cell-inner plexiform layer; GCL=ganglion cell layer; ILM=inner limiting membrane; IPL=inner plexiform layer; m=macular.

For peer review only

Table 2. Demographics, cognitive scores, *APOE* genotype, self-reported comorbidities, medication use and availability of eye imaging factors for UK Biobank participants attending the baseline imaging assessment to-date (*N* = 48,998).

Characteristics	n (%) or mean (SD)
Age (years)	55.2 (7.6)
Sex	
Female	25,290 (52%)
Male	23,708 (48%)
Cognitive scores at baseline assessment	
Numeric memory: maximum digits remembered correctly (n=4,911)	6.97 (1.25)
Fluid intelligence score (n=16,427)	6.68 (2.04)
Prospective memory test (n=16,544)	1.10 (0.36)
Snap game: mean time to correctly identify matches (ms) (n=48,858)	539.2 (101.3)
Pairs matching: number of incorrect matches in round (n=24,988)	0.66 (1.24)
APOE genotype	
ε3ε3	28,297 (59%)
ε3ε4	11,063 (23%)
ε2ε3	5,892 (12%)
ε2ε4	1,128 (2%)
ε4ε4	1,065 (2%)
ε2ε2	277 (1%)
Comorbidities	
Hypertension	13,666 (28%)
Diabetes	2,008 (4%)
Ischemic heart disease	2,649 (5%)
Stroke	719 (1%)
Chronic obstructive pulmonary disease	1,016 (2%)
Asthma	6,689 (14%)
Obesity (BMI > 30 kg/m^2)	8,918 (18%)
Parkinson's disease	136 (<1%)
Alzheimer's disease	34 (<1%)
Multiple sclerosis	202 (<1%)
Medication use	
Anti-hypertensive	9,769 (20%)
Statin	8,750 (18%)
Eye imaging available	13,732 (28%)

Mean (SD) is presented for continuous variables and count (%) for categorical variables. All variables are presented for the full sample except for APOE genotype (1276 missing) and for cognitive scores (numbers of participants for each test at ANY PHASE of UK Biobank examinations are presented in the table). BMI=body mass index; SD=standard deviation; ms=microsecond; APOE=Apolipoprotein E.

For peer review only

Table 3. Cognitive function measures currently available in UK Biobank at different time points.

	Study phase (n)				
(variable ID)	Baseline (n) (2006-2010)	Repeat Assessment (2012-2013)	Online (2015)	Imaging study (2014-now)	Repeat Imaging (2019-2020)
Fluid IQ (100027)	165,500	20,100	123,500	39,600	800
Pairs matching (100030)	497,900	20,300	118,500	40,400	800
Prospective memory (100031)	171,600	20,300	0	40,400	800
Reaction time (100032)	496,700	20,300	0	40,200	800
Numeric memory (100029)	51,800	0	111,000	28,7000	800
Matrix (501)	0	0	0	27,600	800
Symbol digit substitution (502)	0	0	118,500	27,600	800
Tower test (503)	0	0	0	27,300	800
Picture vocabulary (504)	0	0	0	27,500	800
Trail making (505)	0	0	120,500	27,900	800
Paired associate learning (506)	0	0	0	27,900	800

For peer review only

REFERENCES

1. Reitz C, Mayeux R: **Alzheimer disease: epidemiology, diagnostic criteria, risk factors and biomarkers**. *Biochemical pharmacology* 2014, **88**(4):640-651.
2. Bateman RJ, Xiong C, Benzinger TL, Fagan AM, Goate A, Fox NC, Marcus DS, Cairns NJ, Xie X, Blazey TM: **Clinical and biomarker changes in dominantly inherited Alzheimer's disease**. *N Engl J Med* 2012, **367**:795-804.
3. Tahami Monfared AA, Byrnes MJ, White LA, Zhang Q: **Alzheimer's Disease: Epidemiology and Clinical Progression**. *Neurol Ther* 2022, **11**(2):553-569.
4. Kalaria RN, Maestre GE, Arizaga R, Friedland RP, Galasko D, Hall K, Luchsinger JA, Ogunniyi A, Perry EK, Potocnik F: **Alzheimer's disease and vascular dementia in developing countries: prevalence, management, and risk factors**. *The Lancet Neurology* 2008, **7**(9):812-826.
5. Collaborators G: **Global, regional, and national burden of Alzheimer's disease and other dementias, 1990-2016: a systematic analysis for the Global Burden of Disease Study 2016**. *Lancet Neurol* 2019, **18**(1):88-106.
6. Jack Jr CR, Bennett DA, Blennow K, Carrillo MC, Dunn B, Haeberlein SB, Holtzman DM, Jagust W, Jessen F, Karlawish J: **NIA-AA research framework: toward a biological definition of Alzheimer's disease**. *Alzheimer's & Dementia* 2018, **14**(4):535-562.
7. Mehta D, Jackson R, Paul G, Shi J, Sabbagh M: **Why do trials for Alzheimer's disease drugs keep failing? A discontinued drug perspective for 2010-2015**. *Expert opinion on investigational drugs* 2017, **26**(6):735-739.
8. Gómez-Isla T, Price JL, McKeel Jr DW, Morris JC, Growdon JH, Hyman BT: **Profound loss of layer II entorhinal cortex neurons occurs in very mild Alzheimer's disease**. *Journal of Neuroscience* 1996, **16**(14):4491-4500.
9. Long JM, Holtzman DM: **Alzheimer disease: an update on pathobiology and treatment strategies**. *Cell* 2019, **179**(2):312-339.
10. Dubois B, Villain N, Frisoni GB, Rabinovici GD, Sabbagh M, Cappa S, Bejanin A, Bombois S, Epelbaum S, Teichmann M: **Clinical diagnosis of Alzheimer's disease: recommendations of the International Working Group**. *The Lancet Neurology* 2021, **20**(6):484-496.
11. Cheung CY-l, Ikram MK, Sabanayagam C, Wong TY: **Retinal microvasculature as a model to study the manifestations of hypertension**. *Hypertension* 2012, **60**(5):1094-1103.
12. Lesage S, Mosley T, Wong T, Szklo M, Knopman D, Catellier DJ, Cole S, Klein R, Coresh J, Coker L: **Retinal microvascular abnormalities and cognitive decline: the ARIC 14-year follow-up study**. *Neurology* 2009, **73**(11):862-868.
13. Petzold A, Balcer LJ, Calabresi PA, Costello F, Frohman TC, Frohman EM, Martinez-Lapiscina EH, Green AJ, Kardon R, Outteryck O: **Retinal layer segmentation in multiple sclerosis: a systematic review and meta-analysis**. *The Lancet Neurology* 2017, **16**(10):797-812.
14. O'Bryhim BE, Apte RS, Kung N, Coble D, Van Stavern GP: **Association of preclinical Alzheimer disease with optical coherence tomographic angiography findings**. *JAMA ophthalmology* 2018, **136**(11):1242-1248.
15. Mutlu U, Colijn JM, Ikram MA, Bonnemaijer PW, Licher S, Wolters FJ, Tiemeier H, Koudstaal PJ, Klaver CC, Ikram MK: **Association of retinal neurodegeneration on**

For peer review only

optical coherence tomography with dementia: a population-based study. *JAMA neurology* 2018, **75**(10):1256-1263.
16. Masuzzo A, Dinet V, Cavanagh C, Mascarelli F, Krantic S: **Amyloidosis in retinal neurodegenerative diseases**. *Frontiers in Neurology* 2016, **7**:127.
17. Reitz C, Brayne C, Mayeux R: **Epidemiology of Alzheimer disease**. *Nature Reviews Neurology* 2011, **7**(3):137-152.
18. Lambert J-C, Ibrahim-Verbaas CA, Harold D, Naj AC, Sims R, Bellenguez C, Jun G, DeStefano AL, Bis JC, Beecham GW: **Meta-analysis of 74,046 individuals identifies 11 new susceptibility loci for Alzheimer's disease**. *Nature genetics* 2013, **45**(12):1452-1458.
19. Norton S, Matthews FE, Barnes DE, Yaffe K, Brayne C: **Potential for primary prevention of Alzheimer's disease: an analysis of population-based data**. *The Lancet Neurology* 2014, **13**(8):788-794.
20. Larsson SC, Traylor M, Malik R, Dichgans M, Burgess S, Markus HS: **Modifiable pathways in Alzheimer's disease: Mendelian randomisation analysis**. *bmj* 2017, **359**.
21. Bowen M, Zutshi H, Cordiner M, Crutch S, Shakespeare T: **Qualitative, exploratory pilot study to investigate how people living with posterior cortical atrophy, their carers and clinicians experience tests used to assess vision**. *BMJ open* 2019, **9**(3):e020905.
22. Chua SYL, Thomas D, Allen N, Lotery A, Desai P, Patel P, Muthy Z, Sudlow C, Peto T, Khaw PT: **Cohort profile: design and methods in the eye and vision consortium of UK Biobank**. *BMJ open* 2019, **9**(2):e025077.
23. Littlejohns TJ, Sudlow C, Allen NE, Collins R: **UK Biobank: opportunities for cardiovascular research**. *European heart journal* 2019, **40**(14):1158-1166.
24. Bycroft C, Freeman C, Petkova D, Band G, Elliott LT, Sharp K, Motyer A, Vukcevic D, Delaneau O, O'Connell J: **The UK Biobank resource with deep phenotyping and genomic data**. *Nature* 2018, **562**(7726):203-209.
25. Littlejohns TJ, Holliday J, Gibson LM, Garratt S, Oesingmann N, Alfaro-Almagro F, Bell JD, Boultwood C, Collins R, Conroy MC: **The UK Biobank imaging enhancement of 100,000 participants: rationale, data collection, management and future directions**. *Nature communications* 2020, **11**(1):1-12.
26. Huber R, Wojtkowski M, Fujimoto JG, Jiang J, Cable A: **Three-dimensional and C-mode OCT imaging with a compact, frequency swept laser source at 1300 nm**. *Optics express* 2005, **13**(26):10523-10538.
27. Keane PA, Grossi CM, Foster PJ, Yang Q, Reisman CA, Chan K, Peto T, Thomas D, Patel PJ, Consortium UBEV: **Optical coherence tomography in the UK biobank study–rapid automated analysis of retinal thickness for large population-based studies**. *PLoS One* 2016, **11**(10):e0164095.
28. Cruz-Herranz A, Balk LJ, Oberwahrenbrock T, Saidha S, Martinez-Lapiscina EH, Lagreze WA, Schuman JS, Villoslada P, Calabresi P, Balcer L: **The APOSTEL recommendations for reporting quantitative optical coherence tomography studies**. *Neurology* 2016, **86**(24):2303-2309.
29. Welikala R, Fraz M, Habib M, Daniel-Tong S, Yates M, Foster P, Whincup P, Rudnicka AR, Owen CG, Strachan D: **Automated quantification of retinal vessel morphometry in the UK Biobank Cohort**. In: *2017 Seventh International Conference on Image Processing Theory, Tools and Applications (IPTA): 2017*: IEEE; 2017: 1-6.

For peer review only - http://bmjopen.bmj.com/site/about/guidelines.xhtml

30. Welikala R, Fraz M, Foster P, Whincup P, Rudnicka AR, Owen CG, Strachan D, Barman SA: **Automated retinal image quality assessment on the UK Biobank dataset for epidemiological studies**. *Computers in biology and medicine* 2016, **71**:67-76.

31. Fraz MM, Welikala R, Rudnicka AR, Owen CG, Strachan D, Barman SA: **QUARTZ: Quantitative Analysis of Retinal Vessel Topology and size–an automated system for quantification of retinal vessels morphology**. *Expert Systems with Applications* 2015, **42**(20):7221-7234.

32. Welikala R, Foster P, Whincup P, Rudnicka AR, Owen CG, Strachan D, Barman S: **Automated arteriole and venule classification using deep learning for retinal images from the UK Biobank cohort**. *Computers in biology and medicine* 2017, **90**:23-32.

33. Tapp RJ, Owen CG, Barman SA, Welikala RA, Foster PJ, Whincup PH, Strachan DP, Rudnicka AR, UK Biobank Eye VC: **Retinal vascular tortuosity and diameter associations with adiposity and components of body composition**. *Obesity* 2020, **28**(9):1750-1760.

34. Tapp RJ, Owen CG, Barman SA, Welikala RA, Foster PJ, Whincup PH, Strachan DP, Rudnicka AR: **Associations of Retinal Microvascular Diameters and Tortuosity With Blood Pressure and Arterial Stiffness: United Kingdom Biobank**. *Hypertension* 2019, **74**(6):1383-1390.

35. Rudnicka AR, Welikala R, Barman S, Foster PJ, Luben R, Hayat S, Khaw KT, Whincup P, Strachan D, Owen CG: **Artificial intelligence-enabled retinal vasculometry for prediction of circulatory mortality, myocardial infarction and stroke**. *Br J Ophthalmol* 2022.

36. McGrory S, Taylor AM, Pellegrini E, Ballerini L, Kirin M, Doubal FN, Wardlaw JM, Doney AS, Dhillon B, Starr JM: **Towards standardization of quantitative retinal vascular parameters: comparison of SIVA and VAMPIRE measurements in the Lothian Birth Cohort 1936**. *Translational vision science & technology* 2018, **7**(2):12-12.

37. Perez-Rovira A, MacGillivray T, Trucco E, Chin K, Zutis K, Lupascu C, Tegolo D, Giachetti A, Wilson PJ, Doney A: **VAMPIRE: vessel assessment and measurement platform for images of the REtina**. In: *2011 Annual International Conference of the IEEE Engineering in Medicine and Biology Society: 2011*: IEEE; 2011: 3391-3394.

38. Remond P, Aptel F, Cunnac P, Labarere J, Palombi K, Pepin J-L, Pollet-Villard F, Hogg S, Wang R, MacGillivray T: **Retinal vessel phenotype in patients with nonarteritic anterior ischemic optic neuropathy**. *American journal of ophthalmology* 2019, **208**:178-184.

39. Azanan MS, Chandrasekaran S, Rosli ES, Chua LL, Oh L, Chin TF, Yap TY, Rajagopal R, Rajasuriar R, MacGillivray T: **Retinal Vessel Analysis as a Novel Screening Tool to Identify Childhood Acute Lymphoblastic Leukemia Survivors at Risk of Cardiovascular Disease**. *Journal of pediatric hematology/oncology* 2020, **42**(6):e394-e400.

40. Ko F, Muthy ZA, Gallacher J, Sudlow C, Rees G, Yang Q, Keane PA, Petzold A, Khaw PT, Reisman C: **Association of retinal nerve fiber layer thinning with current and future cognitive decline: a study using optical coherence tomography**. *JAMA neurology* 2018, **75**(10):1198-1205.

41. Cornelis MC, Wang Y, Holland T, Agarwal P, Weintraub S, Morris MC: **Age and cognitive decline in the UK Biobank**. *PloS one* 2019, **14**(3):e0213948.

For peer review only - http://bmjopen.bmj.com/site/about/guidelines.xhtml

42. Chua SY, Lascaratos G, Atan D, Zhang B, Reisman C, Khaw PT, Smith SM, Matthews PM, Petzold A, Strouthidis NG: **Relationships between retinal layer thickness and brain volumes in the UK Biobank cohort**. *European Journal of Neurology* 2021, **28**(5):1490-1498.

43. Lyall DM, Cullen B, Allerhand M, Smith DJ, Mackay D, Evans J, Anderson J, Fawns-Ritchie C, McIntosh AM, Deary IJ: **Cognitive test scores in UK Biobank: data reduction in 480,416 participants and longitudinal stability in 20,346 participants**. *PloS one* 2016, **11**(4):e0154222.

44. Riley RD, Snell KI, Ensor J, Burke DL, Harrell Jr FE, Moons KG, Collins GS: **Minimum sample size for developing a multivariable prediction model: PART II-binary and time-to-event outcomes**. *Statistics in medicine* 2019, **38**(7):1276-1296.

45. Petermann-Rocha F, Lyall DM, Gray SR, Esteban-Cornejo I, Quinn TJ, Ho FK, Pell JP, Celis-Morales C: **Associations between physical frailty and dementia incidence: a prospective study from UK Biobank**. *The Lancet Healthy Longevity* 2020, **1**(2):e58-e68.

46. Standard B: **Test charts for determining distance visual acuity: BS 4274-1968**. *British Standards Institute* 1968.

47. Tikkanen E, Gustafsson S, Ingelsson E: **Associations of fitness, physical activity, strength, and genetic risk with cardiovascular disease: longitudinal analyses in the UK Biobank Study**. *Circulation* 2018, **137**(24):2583-2591.

48. Perez-Cornago A, Key TJ, Allen NE, Fensom GK, Bradbury KE, Martin RM, Travis RC: **Prospective investigation of risk factors for prostate cancer in the UK Biobank cohort study**. *British journal of cancer* 2017, **117**(10):1562-1571.

49. Allen NE, Sudlow C, Peakman T, Collins R, biobank U: **UK biobank data: come and get it**. In., vol. 6: American Association for the Advancement of Science; 2014: 224ed224-224ed224.

50. Ntuk UE, Gill JM, Mackay DF, Sattar N, Pell JP: **Ethnic-specific obesity cutoffs for diabetes risk: cross-sectional study of 490,288 UK biobank participants**. *Diabetes care* 2014, **37**(9):2500-2507.

51. Millett ER, Peters SA, Woodward M: **Sex differences in risk factors for myocardial infarction: cohort study of UK Biobank participants**. *bmj* 2018, **363**.

52. Gallacher KI, McQueenie R, Nicholl B, Jani BD, Lee D, Mair FS: **Risk factors and mortality associated with multimorbidity in people with stroke or transient ischaemic attack: a study of 8,751 UK Biobank participants**. *Journal of comorbidity* 2018, **8**(1):1-8.

53. Ma H, Li X, Sun D, Zhou T, Ley SH, Gustat J, Heianza Y, Qi L: **Association of habitual glucosamine use with risk of cardiovascular disease: prospective study in UK Biobank**. *bmj* 2019, **365**.

54. Gao L, Li P, Cui L, Wong PM, Johnson-Akeju O, Lane J, Saxena R, Scheer F, Hu K: **Sleep disturbance and incident Alzheimer’s disease: A UK Biobank study of 502,538 middle-aged to older participants: Biomarkers (non-neuroimaging): Alzheimer's disease incidence, risk factors and biomarkers**. *Alzheimer's & Dementia* 2020, **16**:e044575.

55. Veronese N, Yang L, Piccio L, Smith L, Firth J, Marx W, Giannelli G, Caruso MG, Cisternino AM, Notarnicola M: **Adherence to a healthy lifestyle and multiple sclerosis: a case–control study from the UK Biobank**. *Nutritional neuroscience* 2020:1-9.

For peer review only - http://bmjopen.bmj.com/site/about/guidelines.xhtml

56. Petzold A, Chua SY, Khawaja AP, Keane PA, Khaw PT, Reisman C, Dhillon B, Strouthidis NG, Foster PJ, Patel PJ: **Retinal asymmetry in multiple sclerosis**. *Brain* 2021, **144**(1):224-235.
57. Petzold A, Fraser CL, Abegg M, Alroughani R, Alshowaeir D, Alvarenga R, Andris C, Asgari N, Barnett Y, Battistella R: **Diagnosis and classification of optic neuritis**. *The Lancet Neurology* 2022.
58. Cumberland PM, Rahi JS: **Visual function, social position, and health and life chances: the UK biobank study**. *JAMA ophthalmology* 2016, **134**(9):959-966.
59. Chan MP, Grossi CM, Khawaja AP, Yip JL, Khaw K-T, Patel PJ, Khaw PT, Morgan JE, Vernon SA, Foster PJ: **Associations with intraocular pressure in a large cohort: results from the UK Biobank**. *Ophthalmology* 2016, **123**(4):771-782.
60. Shah RL, Guggenheim JA: **Genome-wide association studies for corneal and refractive astigmatism in UK Biobank demonstrate a shared role for myopia susceptibility loci**. *Human genetics* 2018, **137**(11):881-896.
61. Wood A, Guggenheim JA: **Refractive error has minimal influence on the risk of age-related macular degeneration: a Mendelian randomization study**. *American journal of ophthalmology* 2019, **206**:87-93.
62. Craig JE, Han X, Qassim A, Hassall M, Cooke Bailey JN, Kinzy TG, Khawaja AP, An J, Marshall H, Gharahkhani P: **Multitrait analysis of glaucoma identifies new risk loci and enables polygenic prediction of disease susceptibility and progression**. *Nature genetics* 2020, **52**(2):160-166.
63. de Jong FJ, Schrijvers EM, Ikram MK, Koudstaal PJ, de Jong PT, Hofman A, Vingerling JR, Breteler MM: **Retinal vascular caliber and risk of dementia: the Rotterdam study**. *Neurology* 2011, **76**(9):816-821.
64. Mutlu U, Cremers LG, De Groot M, Hofman A, Niessen WJ, Van Der Lugt A, Klaver CC, Ikram MA, Vernooij MW, Ikram MK: **Retinal microvasculature and white matter microstructure: the Rotterdam Study**. *Neurology* 2016, **87**(10):1003-1010.
65. Mutlu U, Bonnemaijer PW, Ikram MA, Colijn JM, Cremers LG, Buitendijk GH, Vingerling JR, Niessen WJ, Vernooij MW, Klaver CC: **Retinal neurodegeneration and brain MRI markers: the Rotterdam Study**. *Neurobiology of aging* 2017, **60**:183-191.
66. Sudlow C, Gallacher J, Allen N, Beral V, Burton P, Danesh J, Downey P, Elliott P, Green J, Landray M: **UK biobank: an open access resource for identifying the causes of a wide range of complex diseases of middle and old age**. *PLoS medicine* 2015, **12**(3):e1001779.
67. Biobank U: **UK Biobank ethics and governance framework**. In.; 2015.

For peer review only - http://bmjopen.bmj.com/site/about/guidelines.xhtml
